# Alterations of c‐di‐GMP turnover proteins modulate semi‐constitutive rdar biofilm formation in commensal and uropathogenic *Escherichia coli*

**DOI:** 10.1002/mbo3.508

**Published:** 2017-09-15

**Authors:** Annika Cimdins, Roger Simm, Fengyang Li, Petra Lüthje, Kaisa Thorell, Åsa Sjöling, Annelie Brauner, Ute Römling

**Affiliations:** ^1^ Department of Microbiology, Tumor, and Cell Biology Karolinska Institutet Stockholm Sweden; ^2^ Norwegian Veterinary Institute Oslo Norway; ^3^ Division of Clinical Microbiology Karolinska University Hospital Stockholm Sweden; ^4^ Present address: Institute of Hygiene University of Münster Münster Germany; ^5^ Present address: Department of Oral Biology Faculty of Dentistry University of Oslo Oslo Norway; ^6^ Present address: Division of Clinical Microbiology Department of Laboratory Medicine Karolinska Institutet and Karolinska University Hospital Huddinge Stockholm Sweden

**Keywords:** c‐di‐GMP, cellulose, CsgD, curli, rdar biofilm, semi‐constitutive biofilm formation

## Abstract

Agar plate‐based biofilm of enterobacteria like *Escherichia coli* is characterized by expression of the extracellular matrix components amyloid curli and cellulose exopolysaccharide, which can be visually enhanced upon addition of the dye Congo Red, resulting in a red, dry, and rough (rdar) colony morphology. Expression of the rdar morphotype depends on the transcriptional regulator CsgD and occurs predominantly at ambient temperature in model strains. In contrast, commensal and pathogenic isolates frequently express the *csgD*‐dependent rdar morphotype semi‐constitutively, also at human host body temperature. To unravel the molecular basis of temperature‐independent rdar morphotype expression, biofilm components and c‐di‐GMP turnover proteins of seven commensal and uropathogenic *E. coli* isolates were analyzed. A diversity within the c‐di‐GMP signaling network was uncovered which suggests alteration of activity of the trigger phosphodiesterase YciR to contribute to (up)regulation of *csgD* expression and consequently semi‐constitutive rdar morphotype development.

## 1 INTRODUCTION

Biofilm formation, per definition “matrix‐enclosed bacterial populations adherent to each other and/or to surfaces or interfaces” (Costerton, Lewandowski, Caldwell, Korber, & Lappin‐Scott, 1995) constitutes the predominant life form of bacteria. The extracellular matrix consists of different components such as fimbriae, exopolysaccharides, extracellular DNA, and proteins. In enterobacteria, the well‐studied red, dry, and rough (rdar) morphotype is a biofilm phenotype characterized by expression of curli fimbriae and cellulose, that can be investigated on LB without salt agar plates supplemented with either Congo Red (CR), which binds to both matrix components or Calcofluor (CF), which binds predominantly to cellulose (Römling, 2005). Sole expression of curli results in a brown, dry, and rough colony (bdar), while cellulose expression leads to a pink‐stained phenotype (pdar) on CR agar plates.

Rdar morphotype expression depends on the master transcriptional regulator CsgD (Gerstel, Kolb, & Römling, 2006; Gerstel & Römling, 2003; Liu, Niu, Wu, & Huang, 2014; Simm, Ahmad, Rhen, Le Guyon, & Römling, 2014). *CsgD* expression itself is regulated at different levels (Bordeau & Felden, 2014; Gerstel & Römling, 2003; Gerstel et al., 2006; Mika & Hengge, 2013; Prigent‐Combaret et al., 2001; Simm et al., 2014). *CsgD* expression is induced in the stationary phase of growth, preferably under low salt condition at ambient temperature, but single point mutations in the *csgD* promoter region can overcome temperature regulation of *csgD* in both *Escherichia coli* (Uhlich, Keen, & Elder, 2001) and *Salmonella enterica* serovar Typhimurium (Römling, Sierralta, Eriksson, & Normark, 1998). The temperature‐regulated *csgD* promoter is under control of the stationary phase sigma factor σ^S^ and several global regulators, among them OmpR (Gerstel & Römling, 2003) and MlrA (Brown et al., 2001; Ogasawara, Yamamoto, & Ishihama, 2010). The secondary messenger bis‐(3′‐5′)‐cyclic dimeric guanosine monophosphate (c‐di‐GMP) plays a crucial role in *csgD* expression and biofilm formation (Römling, Galperin, & Gomelsky, 2013; Simm, Morr, Kader, Nimtz, & Römling, 2004). Low levels of c‐di‐GMP facilitate flagella‐based motility and high levels promote sessility and biofilm formation. Diguanylate cyclases (DGCs), catalytically functional GGDEF domain proteins, synthesize c‐di‐GMP, while c‐di‐GMP is degraded to 5′pGpG by EAL domain phosphodiesterases (PDEs). The c‐di‐GMP network shows spatial and temporal specificity, and *csgD* expression is controlled by a multitude of c‐di‐GMP turnover enzymes in *E. coli* and *S*. Typhimurium (Ahmad, Cimdins, Beske, & Römling, 2017; Kader, Simm, Gerstel, Morr, & Römling, 2006; Simm, Lusch, Kader, Andersson, & Römling, 2007; Sommerfeldt et al., 2009) such as the DGCs YdaM (*E. coli* only), YedQ and YegE and the PDEs YciR and YhjH. Moreover, a multitude of sRNAs were shown to regulate biofilm formation by acting on *csgD* expression or other biofilm‐related genes (Bak et al., 2015; Mika & Hengge, 2013, 2014; Parker, Cureoglu, De Lay, Majdalani, & Gottesman, 2017).

Downstream, CsgD directly controls expression of the curli subunit genes *csgBAC* and indirectly activates cellulose expression via the diguanylate cyclase AdrA (Hammar, Arnqvist, Bian, Olsen, & Normark, 1995; Römling, Rohde, Olsen, Normark, & Reinköster, 2000; Serra, Richter, & Hengge, 2013). Of note, c‐di‐GMP promotes synthesis of the extracellular matrix component poly‐N‐acetylglucosamine (PNAG), which requires the *pgaABCD* operon (Itoh et al., 2008; Steiner, Lori, Boehm, & Jenal, 2013).

The genome of *E. coli* K‐12 MG1655 contains 29 c‐di‐GMP turnover proteins, 12 DGCs and 13 PDEs, including seven proteins with a GGDEF and an EAL domain, and four GGDEF and/or EAL domain proteins with degenerated motifs. The number of c‐di‐GMP turnover proteins can differ among various *E. coli* strains (Povolotsky & Hengge, 2016). Functionality of these additional proteins in biofilm formation has been proven for DgcX in enteroaggregative *E. coli* (Richter, Povolotsky, Wieler, & Hengge, 2014), for PdeY (SfaY) upon overexpression in *Vibrio cholerae* (Sjöström et al., 2009), and for PdeT (VmpA) in *E. coli* O157:H7 strain EDL933 (Branchu et al., 2013).

*E. coli* strains typically colonize the human gastrointestinal tract, being one of the first colonizers. Biofilm formation of *E. coli* in the gut has been reviewed recently (Rossi et al., 2017). Uropathogenic *E. coli* UPEC can also cause intra‐ and extraintestinal diseases such as urinary tract infection (UTI) (Croxen & Finlay, 2010; Croxen et al., 2013; Kaper, Nataro, & Mobley, 2004; Leimbach, Hacker, & Dobrindt, 2013; Nataro & Kaper, 1998). Variations in rdar morphotype formation such as the semi‐constitutive rdar morphotype, that is, expression of the rdar morphotype not only at ambient temperature, but also at 37°C, occur frequently in commensal and pathogenic isolates (Bian, Brauner, Li, & Normark, 2000; Bokranz, Wang, Tschäpe, & Römling, 2005; Cimdins et al., 2017; Da Re & Ghigo, 2006; Hammar, Arnqvist, Bian, Olsen, & Normark, 1995; Kai‐Larsen et al., 2010; Zogaj, Bokranz, Nimtz, & Römling, 2003; Zogaj, Nimtz, Rohde, Bokranz, & Römling, 2001). For example, the Shiga toxin‐producing 2011 German EAEC‐related outbreak strain O104:H4 produces CsgD and curli at 37°C, but is deficient in cellulose expression (Richter et al., 2014). The probiotic *E. coli* strain Nissle 1917 shows semi‐constitutive cellulose expression that is independent of CsgD and the DGC AdrA (Monteiro et al., 2009). UTI isolates display a broad variety in rdar morphotype formation (Bokranz et al., 2005; Kai‐Larsen et al., 2010).

In a previous study, we observed that *E. coli* strains from fecal samples of healthy individuals differed in rdar biofilm regulation and expression of extracellular matrix components (Bokranz et al., 2005). In this study, we determined the molecular basis of semi‐constitutive rdar biofilm formation of three commensal *E. coli* and four UPEC isolates. Our results show an unexpected high diversity of the c‐di‐GMP signaling network in these strains with respect to functionality, number of c‐di‐GMP turnover proteins and single‐amino acid polymorphisms. We report here alterations in the trigger enzyme YciR to differentially affect rdar biofilm formation.

## 2 EXPERIMENTAL PROCEDURES

### 2.1 Bacterial strains and growth conditions

Strains used in this study are listed in Table 1. For cloning purposes, construction of deletion mutants and site‐directed mutagenesis, strains were grown in LB medium under agitation (200 rpm) or on LB agar plates at the indicated temperatures. For phenotypic analysis, strains were grown on LB medium without salt. If appropriate, media was supplemented with 100 μg/ml ampicillin and different concentrations of l‐arabinose, as stated.

**Table 1:** Strains used in the study

Strain	Source	Relevant information	Reference
*E. coli* strains			
Tob1	Feces	commensal strain	(Bokranz et al., 2005)
Tob2		Tob1 Δ*csgD*	(Bokranz et al., 2005)
Tob1 Δ*yciR*		Tob1 *yciR::Cm*	This study
Fec9	Feces	commensal strain, clonal variant of Tob1	(Bokranz et al., 2005)
Fec10	Feces	commensal strain	(Bokranz et al., 2005)
Fec10 Δ*yciR*		Fec10 *yciR::Cm*	This study
Fec12	Feces	commensal strain, clonal variant of Tob1	(Bokranz et al., 2005)
Fec67	Feces	commensal strain	(Bokranz et al., 2005)
Fec101	Feces	commensal strain	(Bokranz et al., 2005)
B‐8638	Blood	urosepsis strain	(Cimdins et al., 2017)
No.12	Urine	pyelonephritis strain	(Kai‐Larsen et al., 2010)
No.12 Δ*csgD*		No.12 *csgD::Cm*	This study
B‐11870	Blood	urosepsis strain	(Cimdins et al., 2017)
80//6	Urine	UTI strain, clonal variant of B‐11870	(Cimdins et al., 2017)
Nissle 1917	Feces	commensal strain	Ardeypharm
*E. coli* K‐12 laboratory strains			
DH5α		F‐ Φ80Δ*lacZ* ΔM15 Δ(*lacZYA‐argF*)*U169 recA1 endA1 hsdR17*(rK‐mK+) *phoA supE44 thi‐1 gyrA96 relA1 λ‐*	Cloning host; laboratory collection
TOP10		F‐ *mcrA* Δ(*mrr*‐*hsdRMS*‐*mcrBC*) Φ80*lacZ*ΔM15 Δ*lacX74 recA1 araD139* Δ(*ara‐leu*)7697 *galU galK rpsL* (StrR) *end A1 nupG*	Cloning host; Invitrogen
*Salmonella enterica serovar* Typhimurium			
MAE108		Swimming negative control	(Rochon & Römling, 2006)

### 2.2 Construction of chromosomal mutants

Construction of deletion mutants was performed via λ red recombination technique (Datsenko & Wanner, 2000). Primers yciR‐FEC10‐KO‐For and yciR‐FEC10‐KO‐Rev (to construct Fec10 ∆*yciR*), primers YciRTob1delfw and YciRTob1delrv (Tob1 Δ*yciR*), and primers csgD_start and csgD_stop (No.12 ∆*csgD*) were used to amplify the chloramphenicol resistance cassette from the pKD3 template flanked by 40 bp homologous sequences. Mutants were selected on 10 μg/ml and subsequently 25 μg/ml chloramphenicol, verified by PCR with primers flanking the replaced open reading frame, and cured of pKD46 by incubation at 42°C.

### 2.3 Cloning procedures and site‐directed mutagenesis

Genes of interest were amplified using primers listed in Table S2 and ligated into the pBAD30 vector via *Xba*I/*Hin*dIII restriction sites. Site‐directed mutagenesis was performed using the primers listed in Table S2 and applying the Quik‐change Kit II (Agilent) according to the manufacturer's protocol. All constructs were verified by sequencing. All plasmids used in the study are listed in Table S1.

### 2.4 Analysis of colony morphology

Expression of the rdar morphotype was visualized on LB without salt plates supplemented with 40 μg/ml Congo Red (Sigma) and 20 μg/ml Coomassie Brilliant Blue G‐250 (Sigma). Upon expression of cellulose and curli fibers, dye binding is staining the bacterial colony. Development of the colony morphology was investigated at 28°C and 37°C and documented by photographing at distinct time points. Strains of interest were streaked for single colonies and 5 μl of a suspension of OD_600_ 5 was spotted onto the agar plates.

For visualization of cellulose expression, strains were spotted onto LB without salt plates supplemented with 50 μg/ml Calcofluor (Fluorescent Brightener 28, Sigma). Fluorescence was observed under UV light of wave length 365 nm.

### 2.5 Motility assay

Half of a single colony was inoculated into an LB plate solidified with 0.3% agar. Swimming motility was investigated after 6 hr at 37°C. The phenotype was documented, using the Bio RAD GelDocXR+ analyzer. Statistical analysis of the results was done by unpaired Student's *t*‐test, using Graph Pad Prism No. 5 software.

### 2.6 Biofilm in glass tubes

Biofilm formation in glass tubes, expressed as adherence to glass and clumping, was observed by growing the respective strains in LB without salt medium under agitation (200 rpm) for 24 hr at 28°C and documented by photographing.

### 2.7 SDS PAGE and Western Blotting

To monitor expression of CsgD, strains were grown on LB agar plates without salt for 16–18 hr. Here 5 mg cells were harvested, suspended in 1x SDS sample buffer and boiled at 95°C for 10 min. Samples were separated on a denaturing SDS PAGE with 4% stacking and 15% resolving gel. Gels were stained for analysis of equal protein content (0.1% Coomassie Brilliant Blue G‐250, 2% (w/v) ortho‐phosphoric acid, 10% (w/v) ammonium sulfate, (Sigma)) or proteins were subsequently transferred onto a PVDF membrane (Immobilon P; Millipore) via semidry western blotting at 120 mA for 1 hr. Detection of CsgD was performed using a 1:3000 diluted primary rabbit polyclonal anti‐*E. coli* CsgD antibody (Monteiro et al., 2009) and a 1:3000 diluted goat anti‐rabbit secondary antibody (Jackson ImmunoResearch). Chemiluminescence was detected via LAS‐1000 detector (Fujifilm) after treatment with Lumi‐Light Western Blotting substrate (Roche).

### 2.8 Phylogenetic analysis

To verify the phylogenetic type of the analyzed *E. coli* strains, *in silico* typing was performed, using primer sequences as described (Clermont, Christenson, Denamur, & Gordon, 2013). Presence of *arp1, chuA, yjaA, TspE4.C2, arp1‐E*, and *trpA‐C* were tested *in silico* and *in vitro* (Bokranz et al., 2005), and strains were sorted into phylogenetic groups accordingly.

For computation of the phylogenetic tree, all the genomes were annotated with the Prokka pipeline (Seemann, 2014). The Gff files derived from Prokka were used as input files for the Roary pan‐genome pipeline (Page et al., 2015), which was used to identify the core genome of the strains. Alignment of the concatenated 2353 core genes was done with MAFFT (Katoh & Standley, 2013) and the tree was computed with FastTree (Price, Dehal, & Arkin, 2009). Evolview (He et al., 2016) was applied for the tree layout. MLST typing was done online using the service provided by the Center for Genomic Epidemiology, Technical University of Denmark, at https://cge.cbs.dtu.dk/services/MLST/#ref04 (Larsen et al., 2012).

To place the strains of interest into the phylogenetic context, representative *E. coli* isolates from each phylogenetic group (Clermont et al., 2013), and/or each pathovar or ecological niche were analyzed. Strains were: phylogenetic group A: K‐12 substr. MG1655, NC_000913.3; HS, NC_009800.1; phylogenetic group B1: SE11, NC_011415.1; phylogenetic group B2: Nissle 1917, NZ_CP007799.1; phylogenetic group F: SMS‐3‐5, NC_010498.1, uropathogens: UTI89 (group B2), NC_007946.1; CFT073 (group B2), NC_004431.1; UMN026 (group D), NC_011751.1; IAI39 (group F), NC_011750.1, ETEC: H10407 (group A), NC_017633.1; E24377A (group B1), NC_009801.1, EAEC: 55989 (group B1), NC_011748.1; 042 (group D), NC_017626.1, EHEC: 2011c‐3493 (group B1), NC_018658.1; EDL933 (group E), NC_002655.2, EIEC: *E. coli* M4163 (group A), DDBJ/EMBL/GenBank JTCN00000000; *E. coli* 4608‐58 (group F), DDBJ/EMBL/GenBank JTCO00000000; EPEC: E2348/69 (group B2), NC_011601.1; RM12579 (group E), NC_017656.1, AIEC: LF82 (group B2), NC_011993.1; NRG 857C (group B2), NC_017634.1, NMEC: S88 (group B2), CU928161.2; CE10 (group F), NC_017646.1, *Shigella flexneri* 2a str. 301, NC_004337.2; *Shigella sonnei* Ss406, NC_007384.1; *Shigella boydii* Sb227, NC_007613.1; *Shigella dysenteriae* Sd197, NC_007606.1

### 2.9 *In silico* identification of c‐di‐GMP metabolizing proteins

For detailed analysis of the genomic sequences, annotation was carried out with the Prokka package (version 1.10), resulting in three gene builds: For gene build 1, only the reference data included within Prokka was considered. For build 2, gene names called from a curated set of Uniprot proteins for *E. coli* K‐12 were combined with the Prokka data for ‘*Escherichia*’; gene build 3 comprises the same approach, including a more stringent e‐value of 1e‐70.

To identify EAL and GGDEF domains in the genomes of interest, the pfam hidden Markov models (HMM) PF00990 (GGDEF) and PF00563 (EAL) were used to perform an HMM search using the HMMer package version 3.1 against the Prokka annotated protein sequences from build 2. Additionally, all known *E. coli* c‐di‐GMP turnover protein sequences (either K‐12 or the respective reference strain for non‐K‐12 strains (Povolotsky & Hengge, 2016), were used as query for an HMM search and comparison to the genome sequences. The genome sequences of the uropathogenic strain UTI89 (DDBJ/EMBL/GenBank accession no. CP000243) and the probiotic strain Nissle 1917 (DDBJ/EMBL/GenBank accession no. CAPM00000000) were included for comparison.

### 2.10 Bioinformatic tools

RAST (Rapid Annotation using Subsystem Technology) server was used for initial annotation and proteome comparison of strains B‐11870 and 80//6 (Aziz et al., 2008; Brettin et al., 2015; Overbeek et al., 2014). Alignments were created and analyzed using AliView version 1.17.1 (Larsson, 2014). MUSCLE http://www.ebi.ac.uk/Tools/msa/muscle/ (Edgar, 2004) was applied for multiple sequence alignment and is implemented in AliView. Prediction of domains was done using SMART (http://smart.embl-heidelberg.de) (Letunic, Doerks, & Bork, 2015; Schultz, Milpetz, Bork, & Ponting, 1998). Integrated genome viewer (IGV) (http://software.broadinstitute.org/software/igv/) was chosen to browse the Prokka annotated genomes (Robinson et al., 2011; Thorvaldsdottir, Robinson, & Mesirov, 2013).

Sequence editor http://www.fr33.net/seqedit.php and Expasy translate tool http://web.expasy.org/translate/ were used to handle sequences.

## 3 RESULTS

### 3.1 Phenotypic analysis of semi‐constitutive rdar expressing commensal and uropathogenic *E. coli* isolates

**Figure 1:**
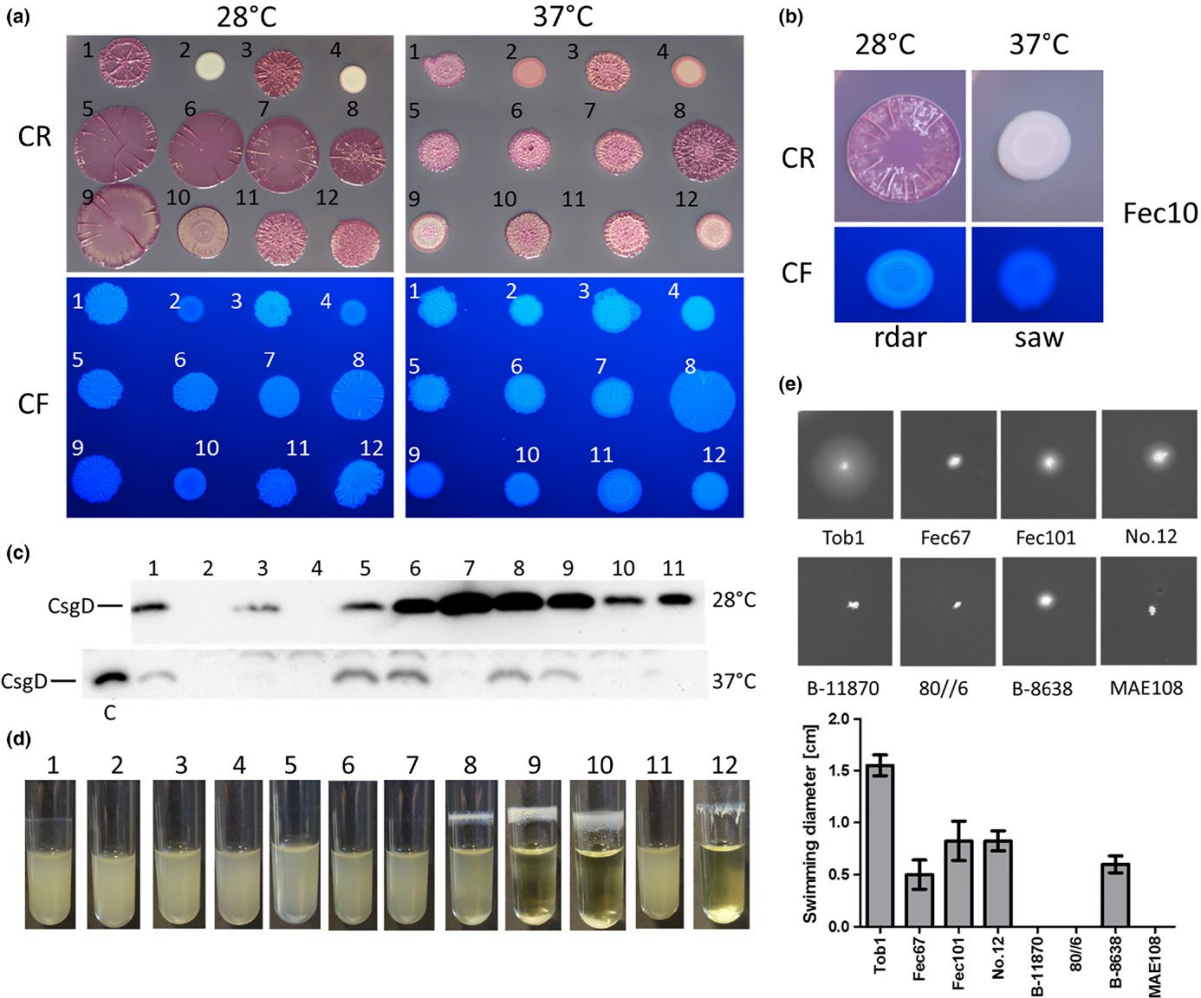
Biofilm formation and swimming motility of semi‐constitutive rdar morphotype forming strains. (a) Rdar morphotype formation on LB without salt agar plates supplemented with 40 μg/ml Congo Red (CR) and 20 μg/ml Coomassie Brilliant Blue G or 50 μg/ml Calcofluor (CF). The strains show semi‐constitutive rdar morphotype formation, apparently expressing curli and cellulose at both 28°C (left) and 37°C (right) after 48 hr incubation. For strains Tob1 and No.12, the morphotype is dependent on CsgD, as respective *∆csgD* mutants are smooth and white at 28°C. *E. coli* Nissle 1917 serves as positive control, exhibiting *csgD*‐independent biofilm formation at 37°C (Da Re & Ghigo, 2006; Monteiro et al., 2009). 1 = Tob1, 2 = Tob1 Δ*csgD*, 3 = No.12, 4 = No.12 Δ*csgD*, 5 = Fec9, 6 = Fec12, 7 = Fec67, 8 = Fec101, 9 = B‐11870, 10 = 80//6, 11 = B‐8638, 12 = Nissle 1917. **(**b) Rdar morphotype formation on LB without salt CR and CF agar plates of strain Fec10 after 48 hr incubation. The strain shows regulated rdar morphotype formation and thus expression of curli and cellulose at 28°C only. (c) CsgD is expressed in all strains grown at 28°C on LB without salt plates, but expression varies at 37°C. Tob1 Δ*csgD* and No.12 Δ*csgD* mutants served as negative control. For comparison, the Tob1 28°C sample was also loaded onto the 37°C gel. 1 = Tob1, 2 = Tob1 Δ*csgD*, 3 = No.12, 4 = No.12 Δ*csgD*, 5 = Fec67, 6 = Fec101, 7 = B‐11870, 8 = 80//6, 9 = B‐8638, 10 = Nissle 1917, 11 = Fec10, C = Tob1 28°C. **(**d) To analyze formation of biofilm on glass under shaking conditions, strains were grown in LB without salt medium at 28°C at 200 rpm for 24 hr. Strains Fec101, B‐11870, 80//6, and Nissle 1917 (positive control) strongly adhered to glass (visible as ring formation) and clumping. The phenotype is much less pronounced for the other strains. Biofilm formation is abolished upon *csgD* deletion in Tob1 and No.12. 1 = Tob1, 2 = Tob1 Δ*csgD*, 3 = No.12, 4 = No.12 Δ*csgD*, 5 = Fec9, 6 =  Fec12, 7 = Fec67, 8 = Fec101, 9 = B‐11870, 10 = 80//6, 11 = B‐8638, 12 = Nissle 1917. (e) Swimming motility at 37°C varies among the strains. Whereas Tob1 exhibits substantial swimming motility, Fec67, Fec101, No.12, and B‐8638 show decreased swimming motility. B‐11870 and 80//6 were nonmotile. MAE108 is a nonmotile *S*. Typhimurium negative control

To investigate the molecular basis of rdar morphotype variability, we selected a panel of seven commensal and uropathogenic *E. coli* strains showing semi‐constitutive rdar morphotype expression (Figure 1a). Although all isolates expressed the rdar morphotype at both 28°C and 37°C, the colony morphology of the strains showed variations in color intensity and roughness (Figure 1a). Strain Fec101 expressed a rugose pattern with relatively low ridges and dark purple color at both 28°C and 37°C. Most of the other strains showed strong rdar morphotype formation at 28°C and less intense coloring, but more structure at 37°C (Figure 1a). An exception is B‐11870, which showed less structure at 37°C (Figure 1a). The probiotic control strain Nissle 1917 (Nissle, 1918) exhibits a pdar morphotype at 37°C, that is cellulose only production, independently of CsgD (Monteiro et al., 2009), while the commensal control strain Fec10 expressed rdar at 28°C only (Figure 1b). As previously shown, the rdar morphotype is dependent on CsgD, as ∆*csgD* mutants of the fecal commensal strain Tob1 and the uropathogenic strain No.12 displayed a smooth and white (saw) morphotype at 28°C (Figure 1a; (Bokranz et al., 2005; Kai‐Larsen et al., 2010)). A smooth and pink colony morphology observed at 37°C indicated basal cellulose expression (Figure 1a). CsgD expression was verified for all semi‐constitutive rdar strains at 28°C. CsgD was detected at 37°C for strains Tob1, Fec67, Fec101, 80//6, and B‐8638, while CsgD levels for the other strains were below the detection limit (Figure 1c), although, of note, *csgD* deletion had an effect in strain No.12 (Figure 1a).

A CsgD‐mediated rdar‐related biofilm phenotype is clumping and adherence to glass in LB without salt medium (Römling, Sierralta, Eriksson, & Normark, 1998). Strains Fec101, B‐11870, 80//6, and the positive control Nissle 1917, formed a ring of adherent bacteria on the glass surface and cells clumped and sedimented at the bottom of the tubes, which resulted in clearance of the medium for the latter three strains at 28°C. Strain Tob1 exhibited low level ring formation that was abolished upon deletion of *csgD* (Figure 1d). At 37°C, strongly diminished or no biofilm formation was observed (data not shown).

Swimming motility, the alternative lifestyle to sessility (i. e., biofilm formation), was tested at 28°C (data not shown) and 37°C. Pronounced motility was observed for Tob1, reduced motility for Fec67, Fec101, No.12, and B‐8638, whereas strains B‐11870 and 80//6 were nonmotile (Figure 1e). Swimming motility was *csgD*‐independent, since *csgD* deletion mutants of strains Tob1 and No.12 displayed similar swimming behavior as the respective wild types (Figure S1), in contrast to a previous study (Dudin, Geiselmann, Ogasawara, Ishihama, & Lacour, 2014).

### 3.2 Phylogenetic typing of *E. coli* strains

**Figure 2:**
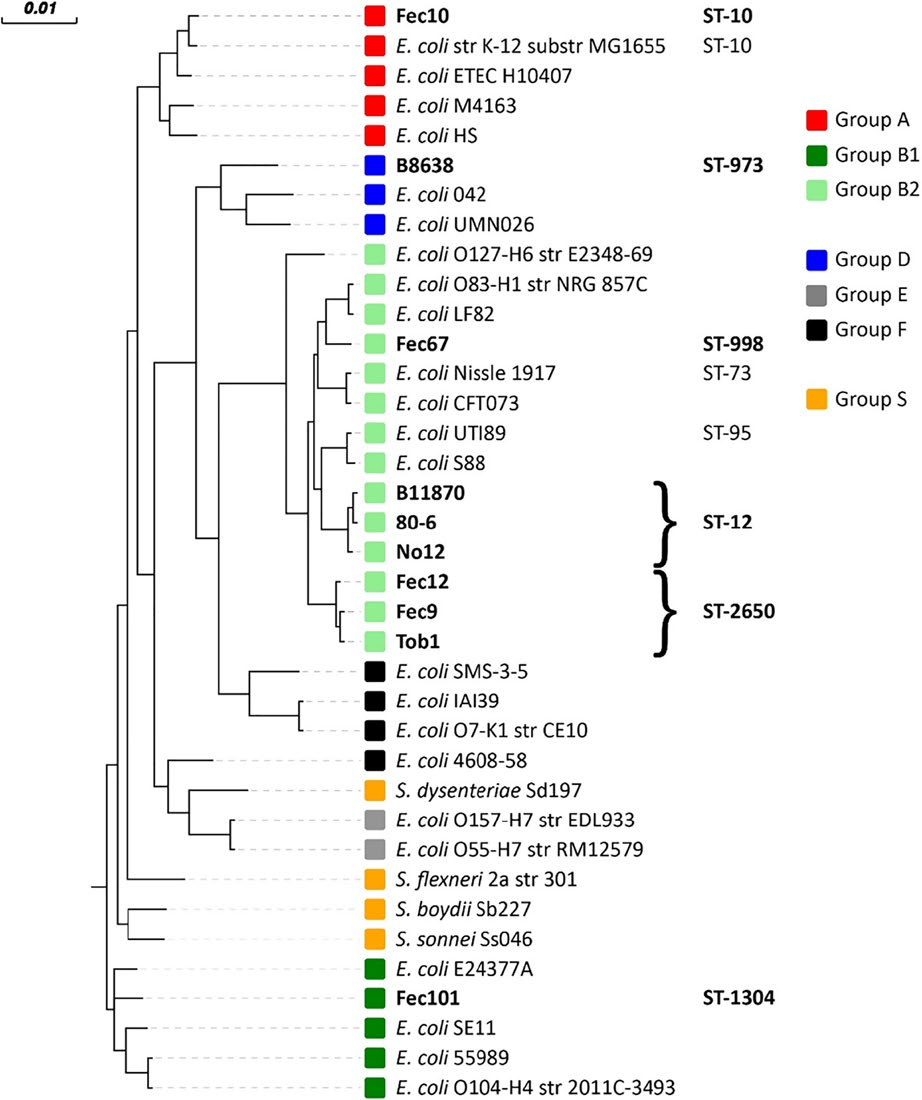
Phylogenetic tree for the semi‐constitutive rdar biofilm strains in relation to phylogenetic groups and *E. coli* pathovars. Core genomes were compared using MAFFT (http://mafft.cbrc.jp/alignment/software/) and the tree was computed by FastTree (http://meta.microbesonline.org/fasttree/). Analysis of the phylo‐groups of the isolates was done by *in silico* testing for the presence of reference genes according to Clermont (Clermont et al., 2013). Multi‐locus sequence typing (MLST) has been applied for identification of the ST‐class. Exemplary strains for different *E. coli* clades and pathovars as well as *Shigella* have been included for comparison. Information about the strains are listed in experimental procedures. Strains analyzed in this study are in bold

Genome sequencing is a powerful tool to gain information about the genetic basis underlying differential biofilm behavior. The genomes of all semi‐constitutive rdar strains in this study have been deposited at DDBJ/ENA/GenBank (Cimdins et al., 2017). Phylogenetic typing, which classifies *E. coli* into 7 phylogroups and *Shigella* phylogroup S, was conducted by *in vitro* and *in silico* PCR (Bokranz et al., 2005; Clermont et al., 2013). This analysis placed strain Fec101 into phylogroup B1, UTI strain B‐8638 into group D, and the remaining semi‐constitutive rdar morphotype expressing UTI and commensal strains into group B2. The commensal strain Fec10 (rdar_28°C_/saw_37°C_) was group A as the reference strain K‐12 MG1655. Subgrouping by *in silico* multilocus sequence typing (MLST) further subclassified the strains into their respective ST‐class (Figure 2; (Larsen et al., 2012)).

### 3.3 Phylogenetic classification o*f E. coli* strains by core genome comparison

In order to get a more detailed phylogenetic classification, the core genomes were compared to a set of reference genomes encompassing representatives from the different *E. coli* phylogenetic groups and pathovars as well as *Shigella* spp. Phylogenetic analyses showed that the commensal isolates Fec12, Fec9, and Tob1 belonged to group B2 and were clonal (Bokranz et al., 2005). In addition, the phylogenetic analyses indicated a close relationship of the uropathogenic strains No.12, B‐11870 and 80//6 despite No.12 being a Slovakian urine isolate, B‐11870 originating from a patient with septicemia in Sweden, and 80//6 originating from a UTI patient in Estonia (Cimdins et al., 2017; Kai‐Larsen et al., 2010). The analysis even implied strains B‐11870 and 80//6 to be clonal variants. A proteome comparison performed with the Rapid Annotation using Subsystem Technology (RAST) server (Aziz et al., 2008; Brettin et al., 2015; Overbeek et al., 2014), indicated an overall protein sequence identity of 98–100% between these two strains. However, insertions of foreign DNA into B‐11870 resulted in a larger genome size of 5.65 Mbp compared to 4.98 Mbp for 80//6 (Cimdins et al., 2017). Nine major insertions of phage‐derived genomes, transposons or plasmid‐related genes, were recognized (Figure S2). Due to their identity in all reference cyclic di‐GMP turnover proteins, further analysis considered only B‐11870. Noteworthy, the commensal strain Fec101 was placed within the same cluster as enteroaggregative strains 55989, 2011C‐3493 (from the 2011 German O104:H4 outbreak incident (Buchholz et al., 2011)), the enterotoxigenic strain E24377A and the environmental strain SE11. These phylogroup B1 strains commonly carry a genomic insertion, including the DGC encoding gene *dgcX* compared to the reference K‐12 core genome (Richter et al., 2014). Only the *dgcX* carrying strain H10407 belongs to phylogroup A.

### 3.4 Strains have a different genomic content of biofilm components

Rdar biofilm formation in *E. coli* is characterized by the presence of four distinct gene modules: (1) two divergently transcribed curli biosynthesis operons *csgBAC* and *csgDEFG* (Hammar et al., 1995; Römling, Bian, Hammar, Sierralta, & Normark, 1998); (2) two divergently transcribed cellulose biosynthesis operons *bcsABZC* and *bcsEFG* (Ahmad et al., 2016; Fang et al., 2014; Römling & Galperin, 2015; Zogaj et al., 2001); (3) distinct c‐di‐GMP turnover proteins required for rdar biofilm formation (Ahmad et al., 2017; Lindenberg, Klauck, Pesavento, Klauck, & Hengge, 2013; Simm et al., 2004), and (4) the *pga* operon coding for poly‐β‐1,6‐N‐acetyl‐D‐glucosamine (PNAG) production, which becomes pronounced upon deletion of the carbon storage regulator CsrA (Itoh et al., 2008).

It is worth noting that the *pga* operon is absent from the genomes of commensal Tob1, Fec101, and UPEC B‐8638. In *E. coli* K‐12 MG1655, the *pgaABCD* operon (complement, 1086106‐1092289) is encoded in vicinity to the *csg* operons (*csgDEFG* complement, 1100851‐1103196; *csgBAC* 1103951‐1105293), directly flanked upstream by oppositely transcribed DGC encoding *ycdT* (see below) and downstream by oppositely transcribed *phoH*, followed by the repeat region REP95 and *efeB*. Directly next to *ycdT*, a mobile element, insertion sequence IS3D, is present (complement 1094245‐1095502) followed by a pseudogene *ymdE* (1095505‐1095846). The next genes in line are *ycdU*,* serX* (complement), *ghrA (ycdW)*,* ycdX*,* ycdY*,* ycdZ*, and the repeat region REP96 (1100821‐1100839) followed by *csgG*.

The *pga* operon is present in the commensal strain Fec67 and UPEC strains B‐11870, 80//6, and No.12, but in a different context compared to K‐12. Although the downstream region with *phoH* is similar, the sequence upstream of *ycdT* deviates from K‐12 MG1655 (Figure S3). In detail, in Fec67, *ycdT* is encoded next to genes of (pro‐)phage origin, genes involved in sugar metabolism (*malY* (maltose regulon modulator; in *E. coli* K‐12 1700957‐1702129), *kbaY* (*E. coli* K‐12 3283143‐3284003), *agaS* (*E. coli* K‐12 3281976‐3283130), *agaR* (*E. coli* K‐12 (complement) 3277856‐3278665), *garD* (*E. coli* K‐12 3275282‐3276853)) and part of a P‐fimbrial gene (coding for PapI). Due to the shotgun nature of the genome sequencing, a more detailed analysis of the genomic context has not been possible, but phage‐derived acquisition of the *pga* operon is indicated.

In strains Tob1, Fec101, and B‐8638, between *phoH* and *csgG*, neither the DGC *ycdT,* the *pga* operon and the insertion element are present, although common with K‐12, *serX*,* ghrA (ycdW)*,* ycdX*,* ycdY*, and *ycdZ* can be found.

The curli and cellulose biosynthesis operons are present in all strains. Single point mutations within the *csgD* promoter have been demonstrated to mediate a semi‐constitutive rdar morphotype both in *S*. Typhimurium (Römling, Sierralta, Eriksson, & Normark, 1998) and *E. coli* (Uhlich et al., 2001). In *E. coli*, these mutations occur within the *csgD* ‐10 promoter region. However, up to ‐41 bps, the *csgD* promoter sequences do not exhibit any point mutations (data not shown). Thus, we reasoned that alterations in the c‐di‐GMP signaling network might contribute to the semi‐constitutive rdar biofilm expression in these commensal and UPEC *E. coli* strains.

### 3.5 Presence and sequence of c‐di‐GMP metabolizing proteins differ among the strains

To investigate the molecular mechanisms of semi‐constitutive rdar morphotype formation, the genome sequences (Cimdins et al., 2017) were first analyzed for the presence or absence of DGCs and PDEs (Table 2). Subsequently, the amino acid (aa) sequences of these proteins were compared to the *E. coli* K‐12 MG1655 reference protein set (Table S3). The non‐K‐12 c‐di‐GMP turnover proteins from the *E. coli* pangenome were also included (Hengge et al., 2015).

Overall, strains showed variability in the number of c‐di‐GMP turnover proteins (Table 2). The vast majority of the known DGCs and PDEs from the *E. coli* pangenome (Hengge et al., 2015; Povolotsky & Hengge, 2016) were present among the strains, but loss of functionality was frequently predicted. Briefly, the DGCs YcdT, and YddV/DosC are not present in three and four strains, respectively. Moreover, stop codons and frame‐shift mutations are present within several open reading frames (ORFs), resulting in potentially nonfunctional truncated proteins. As non‐K‐12 c‐di‐GMP turnover proteins the DGC DgcX in strain Fec101, the EAL protein PdeX in strain Tob1 (99% identity to UTI strain 536 (Povolotsky & Hengge, 2016)) and PdeY in strains No.12, B‐11870 and Fec67 were identified. Full‐length PdeY, described previously for UPEC *E. coli* (Povolotsky & Hengge, 2016; Sjöström et al., 2009), was found in UPEC strains No.12 and B‐11870. Similarly, two truncated versions of PdeY were found in the commensal strain Fec67. In addition, we identified three novel EAL domain proteins PdeU1, PdeU2, and PdeU3 in UPEC strains (Table 2); PdeU1 was present in B‐11870 and 80//6, PdeU2 was found in B‐11870, and PdeU3 in B‐8638. Extended conserved aa signatures are critical for PDE activity (Liang, 2015; Römling, Liang, & Dow, 2017; Römling et al., 2013). Conservation of the signature motifs of catalytic residues indicated that only PdeU1 is potentially active (Figure S4). The EAL domain of PdeU2 is truncated, matching only the EGVE and QG motifs, while PdeU3 is lacking the EGVE and QG motifs (Figure S4B).

**Table 2:**
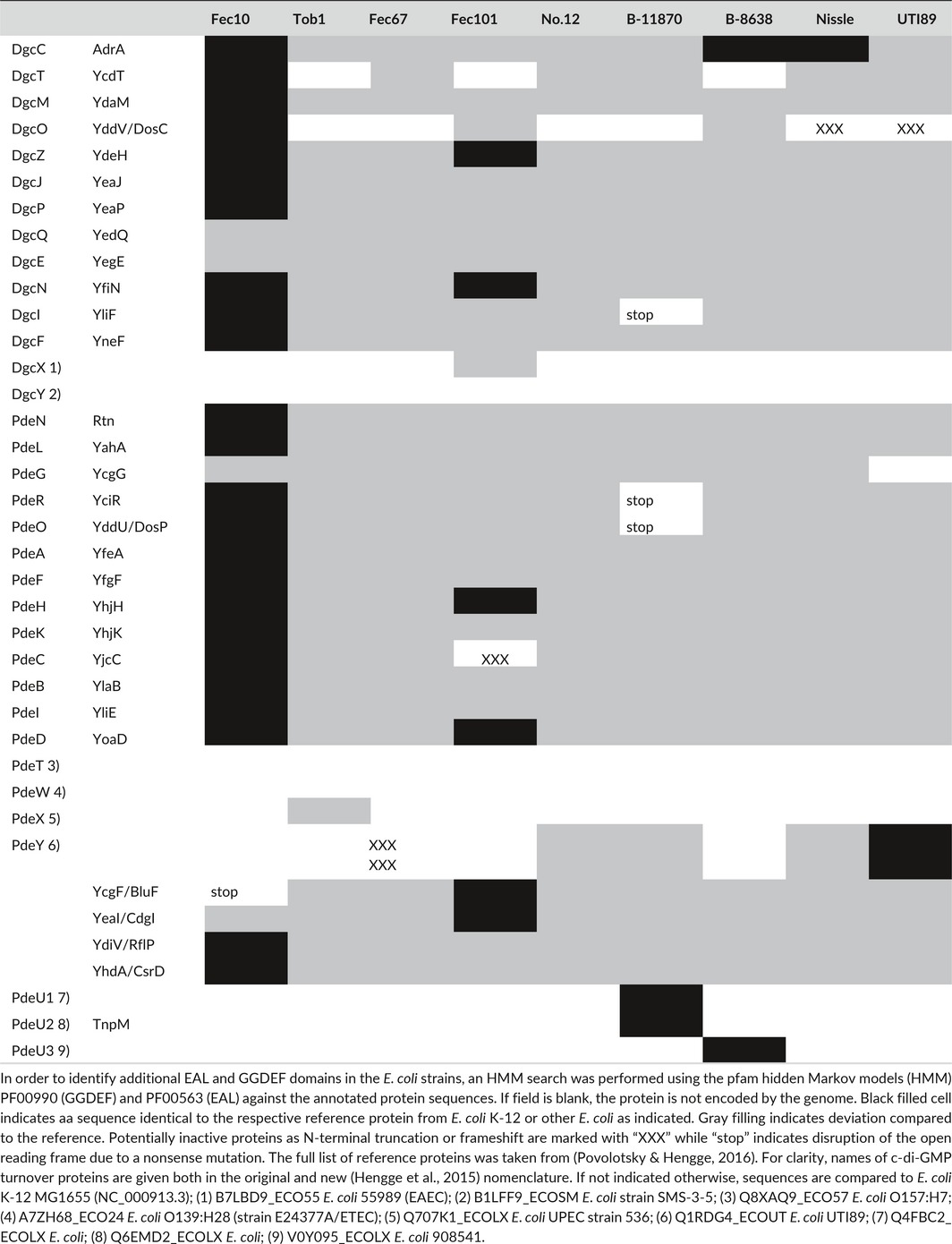
Identification and comparison of c‐di‐GMP metabolizing proteins

Strain Fec10, consistent with its close relatedness to K‐12 and rdar morphotype expression at 28°C, but not 37°C (Figure 1b), encodes all K‐12 c‐di‐GMP turnover proteins, with only five proteins displaying minor aa differences. Among the semi‐constitutive rdar strains, Fec101 is closest to K‐12 with respect to the c‐di‐GMP network proteins, with five proteins to show a 100% identical aa sequence to K‐12, while only one or a few aa are substituted in the other proteins. Fec101, though, encodes additionally the DGC DgcX, lacks the DGC YcdT and harbors a frame‐shift mutation in the PDE encoding gene *yjcC,* that is, the deoxyadenosine monophosphate at nucleotide position 1437 in *yjcC* is deleted. Thus, YjcC_Fec101_ displays a different sequence after aa 479 which eventually results in a truncated EAL domain. Interestingly, in all other semi‐constitutive isolates except B‐8638 nucleotide 1552 is converted from C to T to introduce a TAA stop codon resulting in a 11 aa shorter protein.

The UTI isolate B‐11870 is the strain with the most dissimilar c‐di‐GMP signaling network compared to *E. coli* K‐12. Besides encoding three additional EAL domain proteins, stop codons occur in *yliF*,* yciR*, and the *yddV*/*dosP* PDE encoding gene, with its counterpart the *yddU*/*dosC* DGC encoding gene being completely absent.

We reasoned that specific genes and/or mutations contribute to the semi‐constitutive rdar morphotype behavior. As a first subject of investigation, we focused on the trigger phosphodiesterase YciR, which has previously been shown to be involved in rdar morphotype expression (Hengge, 2016; Lindenberg et al., 2013; Simm et al., 2007).

### 3.6 The trigger enzyme YciR has differential levels of activity among the strains

**Figure 3:**
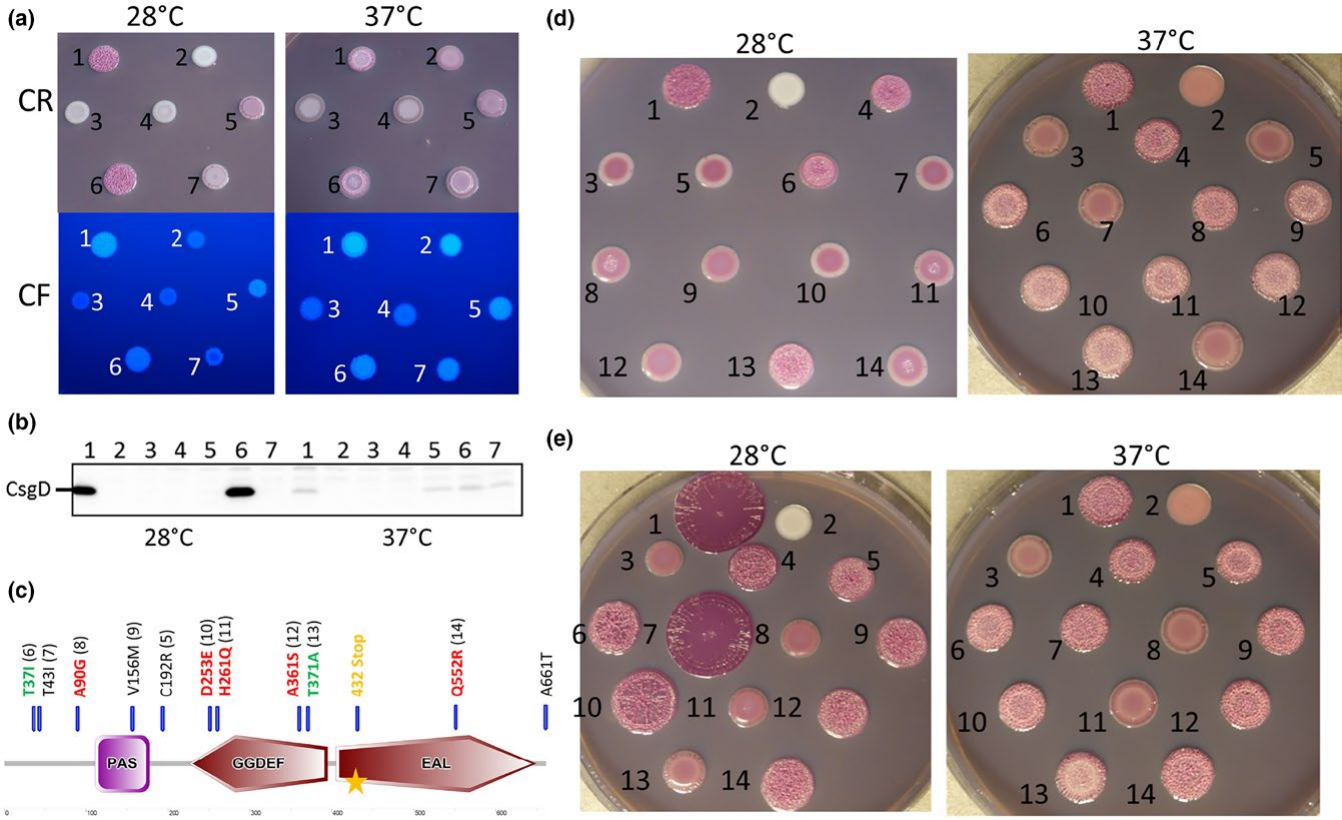
Differential activity of YciR wild type variants from different strains. (a) *E. coli* Tob1 (rdar_28°C_/rdar_37°C_) harbouring plasmid pBAD30 with cloned 6xHis‐YciR (pYciR) from strains Fec10, Fec101, Tob1, B‐11870 and the cloned stop codon revertant YciR_B__‐11870_Full_ grown for 48 hr on CR and CF without salt plates supplemented with 0.1% arabinose at 28°C and 37°C. Overexpression of YciR from Fec10 and Fec101 leads to downregulation of the rdar morphotype at both temperatures, whereas YciR_T__ob1_ exhibits decreased activity. YciR_B__‐11870_ upregulated the rdar morphotype, a regulatory pattern that is reverted upon site directed mutagenesis of the intrinsic TAG stop codon to a TGG sense codon. 1 = Tob1 VC, 2 = Tob1 Δ*csgD* VC, 3 = pYciR_F__ec10_, 4 = pYciR_F__ec101_, 5 = pYciR_T__ob1_, 6 = pYciR_B__‐11870_, 7 = pYciR_B__‐11870_Full_, pYciR=YciR cloned in pBAD30, VC=pBAD30. (b) Western blot detection of CsgD in *E. coli* Tob1 upon expression of 6xHis‐YciR wildtype proteins and the YciR_B__‐11870_Full_ mutant. At 28°C, CsgD expression occurs only for the vector control as well as upon overexpression of YciR_B__‐11870_. At 37°C, CsgD can be detected in the vector control and upon overexpression of YciR_T__ob1_, YciR_B__‐11870_ and YciR_B__‐11870_Full_. 1 = Tob1 VC, 2 = Tob1 Δ*csgD* VC, 3 = pYciR_F__ec10_, 4 = pYciR_F__ec101_, 5 = pYciR_T__ob1_, 6 = pYciR_B__‐11870_, 7 = pYciR_B__‐11870_Full_, pYciR=YciR cloned in pBAD30. VC=pBAD30. (c) Schematic indication of position of noncatalytic aa substitutions referring to the differences between YciR_F__ec10_ and YciR_T__ob1_ and the stop codon in the ORF of YciR_B__‐11870_ (marked by orange star). Red colouring indicates an effect of the mutation only at 37°C, green indicates effects at both 28°C and 37°C. (d) To identify the aa substitutions rendering YciR_T__ob1_ less active than YciR_F__ec10_, site‐directed mutagenesis was performed on pBAD30::6xHis‐YciR_F__ec10_ creating single aa substitutions to approximate to the YciR_T__ob1_ sequence. The effect of overexpression of the respective mutant proteins on rdar morphotype formation was tested in Tob1. Rdar morphotype formation was observed on CR supplemented LB without salt agar plates for 48 hr without inducer at 28°C (left panel) or on plates supplemented with 0.01% arabinose at 37°C (right panel). 1 = Tob1 VC, 2 =  Tob1 Δ*csgD* VC, 3 =  pYciR_F__ec10_, 4 = pYciR_T__ob1_, 5 = pYciR_F__ec101_, 6 = pYciR_F__ec10 T37I_, 7 = pYciR_F__ec10__T43I_, 8 = pYciR_F__ec10 A90G_, 9 = pYciR_F__ec10 V156M_, 10 = pYciR_F__ec10 D253E_, 11 = pYciR_F__ec10 H261Q_, 12 = pYciR_F__ec10 A361S_, 13 = pYciR_F__ec10 T371A_, 14 = pYciR_F__ec10 Q552R_, pYciR=YciR cloned in pBAD30, VC=pBAD30. (e) Tob1 rdar morphotype formation upon combination of aa substitutions. Colony morphology was observed on CR supplemented LB without salt agar plates for 48 hr without inducer at 28°C (left panel) or on plates supplemented with 0.01% arabinose at 37°C (right panel). 1 = Tob1 VC, 2 = Tob1 Δ*csgD* VC, 3 = pYciR_F__ec10_, 4 = pYciR_T__ob1_, 5 = pYciR_F__ec10 T37I_, 6 = pYciR_F__ec10 T371A_, 7 = pYciR_F__ec10 T37I, T371A_, 8 = pYciR_F__ec101_, 9 = pYciR_F__ec101 T37I_, 10 = pYciR_F__ec101__T371A_, 11 = pYciR_F__ec10 V156M_, 12 = pYciR_F__ec101 T37I V156M_, 13 = pYciR_F__ec10 A361S_, 14 = pYciR_F__ec101 T37I A361S_, pYciR=YciR cloned in pBAD30, VC = pBAD30

YciR of the commensal strain Fec10 (rdar_28°C_/saw_37°C_) is identical to K‐12; in contrast YciR exhibits aa substitutions in all semi‐constitutive rdar strains. Strain Fec101 exhibits a single aa substitution, C192R; this mutation is present in all semi‐constitutive rdar strains. Seven aa substitutions are present in YciR_B‐8638_, and eleven in YciR_Tob1_, YciR_Fec67_, and YciR_No.12_. Of note, none of the aa substitutions occurred within the consensus catalytic motifs (Table S3). In strain B‐11870, a nonsense mutation created the truncated YciR_B‐11870_ protein with the N‐terminal sensing domain(s) and an intact GGDEF domain with nine aa substitutions (Figures 3c and S5; Tables 2 and S3). Taken together, we considered YciR a candidate to contribute to semi‐constitutive rdar morphotype expression.

First we assessed the effect of an *yciR* deletion in divergent strain backgrounds. Deletion of *yciR* led to a moderate upregulation of the rdar morphotype in strains Fec10 (Figure S6) and Tob1 (data not shown) as previously reported for other *E. coli* (Lindenberg et al., 2013; Spurbeck, Tarrien, & Mobley, 2012). However, a Fec10 ∆*yciR* mutant displayed a rdar morphotype only at 28°C in contrast to *S*. Typhimurium where, upon *yciR* deletion, *csgD* expression was 10‐fold upregulated at 28°C in combination with temperature‐independent expression at 37°C (Garcia et al., 2004; Simm et al., 2007). Therefore, a strain‐specific rather than a species‐specific effect of *yciR* on rdar morphotype formation is postulated.

To assess functionality, *yciR* from Fec10, Fec101, Tob1, and B‐11870 as representatives of the different classes of YciR proteins (identical to K‐12/ one aa substitution/ most possible number of substitutions/ stop codon) were cloned into pBAD30 with an N‐terminal 6xHis‐tag and expressed in Tob1 (rdar_28°C_/rdar_37°C_) at 28°C and 37°C (Figure 3a). The genes were cloned with one primer set, which corresponds to the wild type C‐terminus of YciR_Tob1_ and YciR_B‐11870_, changing the last aa alanine 661 of YciR_Fec10_ and YciR_Fec101_ to threonine. Overexpression of these constructs in comparison to constructs with the original terminal aa alanine showed no impact on functionality (Figure S5B). In the following, YciR_Fec10__A661T_ and YciR_Fec101__A661T_ are referred to as wild type.

Plasmid‐mediated protein expression can be unphysiologically high which may blur physiological alterations in activity. After testing l‐arabinose concentrations from 0 to 0.1%, activity alterations between YciR variants were most discriminatory observed without arabinose at 28°C whereas addition of an inducer subsequently led to nondiscriminatory downregulation. At 37°C, induction with 0.01% l‐arabinose lead to the best discriminatory results. Overexpression of YciR_Fec10_ and YciR_Fec101_ led to significantly diminished rdar morphotype formation at both 28°C and 37°C concomitant with downregulation of CsgD expression (Figure 3a,b). Although YciR_Tob1_ reduces rdar morphotype expression, the effect is much less pronounced at both 28°C and 37°C, obvious at l‐arabinose concentrations up to 0.1% (Figure 3a and data not shown). To emphasize, the PAS‐PAC‐GGDEF protein YciR_B‐11870_ is active as a DGC at 28°C, as concomitant with the rdar morphotype, CsgD expression was highly upregulated upon overexpression of YciR_B‐11870_. YciR_B‐11870_, though, showed almost no effect on rdar morphotype formation upon overexpression at 37°C. The potential of YciR_B‐11870_ to downregulate the rdar morphotype and CsgD expression could be restored upon reverting the TAG stop codon into a TGG sense codon resulting in full length YciR_B‐11870_Full_ protein (Figure 3a,b). At 37°C, CsgD production was not altered compared to the Tob1 vector control upon overexpression of YciR_B‐11870_ and YciR_B‐11870_Full_ (Figure 3b) suggesting no activity or no expression at this temperature.

Importantly, the effect of YciR on rdar morphotype downregulation was dependent on the position of the tag. Surprisingly, both N‐ and C‐terminally tagged YciR_Fec10_ showed higher effectivity in rdar downregulation than the wild type protein without tag, while the C‐terminal tagged version of YciR_Fec10_ was less effective, but a colony color change was observed. Thus, the tag might stabilize the protein and prevent degradation and otherwise alter activity with respect to rdar morphotype regulation (Figure S5C).

### 3.7 Single aa substitutions of noncatalytic residues affect YciR effectivity

YciR_Tob1_ contains eleven aa substitutions compared to YciR_Fec10_ and exhibits substantially altered activity. Of note, all but two of these mutations occur either in the N‐terminal sensing domain (5) or the GGDEF domain (4). To further investigate the molecular basis of differential activity, YciR_Fec10_ was selected as the basis to individually substitute aas different to YciR_Tob1_ (Figure 3c). The effect of the mutant proteins on rdar morphotype formation was subsequently assessed in Tob1 at 28°C and 37°C. Introduction of mutations T37I and T371A most dramatically diminished the effect of YciR_Fec10_ on rdar morphotype downregulation at 28°C resembling almost YciR_Tob1_, whereas the other protein variants could still downregulate rdar morphotype formation effectively, although to a variable extent (Figure 3d, left panel). At 37°C, more aa substitutions showed a pronounced effect: With the exception of T43I and V156M variants, YciR variants T37I, A90G, A361S and T371A closely resembled YciR_Tob1_, while YciR variants D253E, H261Q and Q552R showed an inconsistent phenotype (Figure 3d, right panel and data not shown).

To investigate potential additive effects of aa substitutions on activity, we introduced V156M, A361S and T371A substitutions into the T37I mutant background of YciR_Fec10_ and YciR_Fec101_ (i.e. YciR_Fec10__C192R_). Again, at 28°C, the phenotypes were clear‐cut (Figure 3e, left panel). Combination of the most prominent variants T37I and T371A in YciR_Fec10__T37I T371A_ resulted in an rdar morphotype appearance close to the Tob1 vector control. Introduction of T37I, T371A, and the combinations T37I V156M and T37I A361S into YciR_Fec101_ led to a colony morphology comparable to YciR_Tob1_ overexpression. In conclusion, at 28°C, T37I is the most determinative aa exchange in combination with T371A. At 37°C, YciR_Fec10_, YciR_Fec101_, and its T43I and V156M mutants effectively downregulated rdar morphotype formation, while D253E, H261Q and Q552R mutants showed an inconsistent phenotype. All other single mutants and the respective double and triple variants resembled YciR_Tob1_ in alteration of the rdar morphotype at 37°C (Figure 3e, right panel and data not shown).

In summary, consistent with the proposed functionality of YciR as a trigger enzyme that regulates *csgD* expression through protein–protein interactions (Hengge, 2016; Lindenberg et al., 2013), aa outside of the catalytic motifs contribute to the activity of YciR and are critical for regulation of rdar biofilm formation. Whereas all besides four introduced aa substitutions hampered the ability of YciR_Fec10_ to downregulate the rdar morphotype at 37°C, only two substitutions proved to be highly relevant at 28°C. The amino acid substitutions presumably affect protein stability, the catalytic activity, cyclic di‐GMP sensing, interaction with other proteins or alter signal sensing.

### 3.8 The mode of action of YciR_Fec101_ and YciR_Tob1_ is independent of catalytically relevant amino acid residues

**Figure 4:**
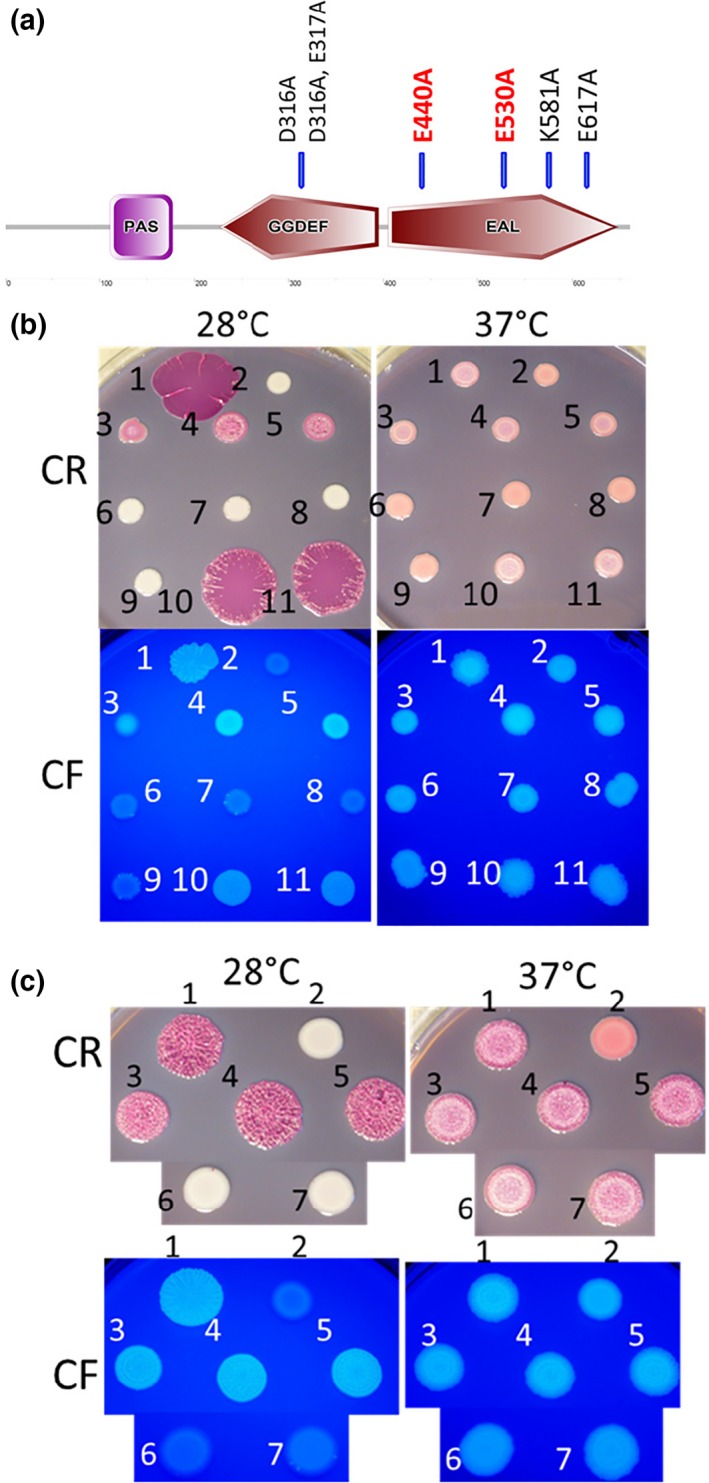
Effects of aa substitutions in the GGDEF and EAL catalytic motifs. (a) Schematic presentation of YciR with indicated substitutions of catalytic aas. Red indicates downregulation of rdar morphotype formation. Domain prediction has been performed with the SMART server. (b) pYciR_F__ec101_ and catalytic mutants expressed in *E. coli* Tob1 (rdar_28°C_/rdar_37°C_). Tob1 harbouring pBAD30 with cloned 6xHis‐YciR_Fec__101_ and respective GGDEF or EAL mutants were grown on CR‐ or CF‐supplemented LB without salt plates at 28°C and 37°C for 48 hr. 1 = Tob1 VC, 2 = Tob1 Δ*csgD* VC, 3 =  pYciR_F__ec101_, 4 =  pYciR_F__ec101 E317A_, 5 = pYciR_F__ec101 D316A, E317A_ 6 = pYciR_F__ec101 E440A_, 7 = pYciR_F__ec101 E317A, E440A_, 8 =  pYciR_F__ec101 D316A, E317A, E440A_ 9 = pYciR_F__ec101 E530A_ 10 = pYciR_F__ec101 K581A_ 11 = pYciR_F__ec101 E617A_ pYciR=YciR cloned in pBAD30, VC=pBAD30. (c) *E. coli* Tob1 colony morphology upon expression of pYciR_Tob1_ wildtype and catalytic mutants at 28°C and 37°C for 48 hr on CR‐ or CF‐supplemented LB without salt plates. 1 = Tob1 VC, 2 = Tob1 Δ*csgD* VC, 3 = pYciR_T__ob1_, 4 =  pYciR_T__ob1 E317A_, 5 = pYciR_T__ob1 D316A, E317A_, 6 = pYciR_T__ob1 E440A_, 7 = pYciR_Tob1 E317A, E440A,_ , pYciR=YciR cloned in pBAD30, VC = pBAD30

YciR is hypothesized to affect *csgD* expression through the interaction with the DGC YdaM and the transcriptional regulator MlrA in response to c‐di‐GMP signaling (Hengge, 2016; Lindenberg et al., 2013). We tested the effect of mutations in the consensus catalytic motifs in YciR_Fec101_ and YciR_Tob1_, being the two extremes in effectiveness on rdar morphotype formation at 28°C and 37°C (Figure 3a). Counterintuitively to its role in catalysis, the E_440_AL to A_440_AL mutant of YciR_Fec101_ (Figure 4b) and YciR_Tob1_ (Figure 4c) downregulated rdar morphotype formation more effectively than the respective wild type proteins. Mutations in the GGD_316_E_317_F motif of YciR_Fec101_ and YciR_Tob1_ resulted in a colony with increased roughness and increased fluorescence under UV light compared to the wild type protein (Figure 4b,c), in contrast to a reported DGC activity of STM1703/YciR, which upregulated CsgD expression in *S*. Typhimurium (Ahmad et al., 2017).

Besides the EAL and GGDEF catalytic motifs, we investigated the impact of aa changes in additional consensus motifs required for the catalytic activity of the EAL domain ((Figure S4) Figure 4b, lowest row). A mutation in the E_530_LTE motif to ALTE downregulated rdar morphotype formation as effectively as the AAL variant. In contrast, expression of YciR_Fec101 K581A_ or YciR_Fec101 E617A_ (E_617_GVE →AGVE) had no effect on rdar morphotype formation.

## 4 DISCUSSION

### 4.1 Rdar morphotype expression is variable among *E. coli* strains

Pathogenic and commensal *E. coli* strains show variability in rdar biofilm formation and regulation (Bokranz et al., 2005; Hammar et al., 1995; Kai‐Larsen et al., 2010; Wang, Lünsdorf, Ehren, Brauner, & Römling, 2010). For example, *E. coli* O157:H7 regularly exhibit a downregulated rdar morphotype and *csgD* expression via either mutation of *rcsB* encoding a response regulator of the Rcs two component system (Sharma et al., 2017), or due to a prophage insertion in the *csgD* specific transcriptional regulator *mlrA* rendering the gene inactive (Chen, Nguyen, Cottrell, Irwin, & Uhlich, 2016). *E. coli* K‐12 derivatives do not produce cellulose due to a stop codon in *bcsQ* (Serra et al., 2013). In this study, we investigated the regulatory basis of semi‐constitutive rdar morphotype expression (Bokranz et al., 2005; Kai‐Larsen et al., 2010).

### 4.2 Rdar morphotype formation is unrelated to the liquid biofilm and motility phenotype

Rdar morphotype formation on CR agar, motility, and biofilm formation in liquid culture cannot be directly correlated. Despite expressing similar rdar morphotypes on CR agar, the strains differed in their ability to clump and adhere to glass when grown in LB without salt broth in shaken liquid cultures. These observations suggest that strain‐specific alterations in the biofilm network dedicated to biofilm formation in liquid culture, potentially in combination with extracellular matrix components alternative to curli and cellulose that cannot be readily observed on CR agar plates, mainly mediate these phenotypes. As such F1C fimbriae are important for biofilm formation of *E. coli* Nissle 1917 in liquid medium (Lasaro et al., 2009). Swimming motility was also variable (Figure 1e) and did not correlate with rdar morphotype expression as observed previously in *Salmonella* spp. (Römling et al., 2003). C‐di‐GMP inhibits motility by binding to the molecular brake protein YcgR (Boehm et al., 2010; Paul, Nieto, Carlquist, Blair, & Harshey, 2010; Ryjenkov, Simm, Römling, & Gomelsky, 2006) and through production of cellulose (Le Guyon, Simm, Rehn, & Römling, 2015; Zorraquino et al., 2013)). Motility is promoted by the key PDE YhjH, which effectively removes motility‐dedicated c‐di‐GMP (Le Guyon et al., 2015; Pesavento et al., 2008; Simm et al., 2007) and consequently the motility/sessility switch is observed upon deletion of YhjH (Simm et al., 2007) or, more pronounced, upon overexpression of DGCs and PDEs (Simm et al., 2004). Although *csgD* represses swimming motility in *E. coli* K‐12 (Dudin et al., 2014; Ogasawara, Yamamoto, & Ishihama, 2011), a correlation between *csgD* expression and motility was not observed. Whether lack of swimming motility in specific strains is due to dysfunctionality of the flagella regulon or differential functionality of c‐di‐GMP turnover proteins has to be further elucidated.

### 4.3 Variability in c‐di‐GMP turnover proteins indicates adaptation to host or environmental conditions

In the rdar_28°C_/saw_37°C_ strain Fec10, a close relative to *E. coli* K‐12 MG1655, all c‐di‐GMP turnover proteins are present and most are identical to MG1655. This is surprising as Fec10 was isolated more than 50 years after *E. coli* K‐12. The other strains contain additional gene products, gene deletions, gene truncations and aa substitutions in their c‐di‐GMP turnover protein network. The GGDEF and EAL domain protein pool analyzed from 61 *E. coli* genomes derived from pathogenic, commensal, or probiotic strains had indicated conserved core reference c‐di‐GMP turnover proteins AdrA, YliF, Rtn, YhjH, YhjK, YlaB, YhdA, YeaI in all strains, suggesting the importance of these proteins under all growth conditions (Povolotsky & Hengge, 2016). However, with the exception of YhjH and YeaI in strain Fec101 and AdrA in B‐8638, all K‐12 reference c‐di‐GMP turnover proteins show aa variations in the semi‐constitutive rdar isolates. Surprisingly, several DGCs seem to be dysfunctional in the semi‐constitutive isolates. For example, the DGC YliF presented a nonsense mutation in strain B‐11870. As reflected by our data, though, the DGCs YcdT and YddV (DosC) were frequently absent independently of strain origin (Povolotsky & Hengge, 2016). Strains lacking DosC and/or DosP might not fully respond to oxygen to regulate biofilm formation, as the DosC/P system was shown to regulate curli and PNAG expression in response to oxygen levels (Jonas et al., 2010; Tagliabue, Maciag, Antoniani, & Landini, 2010; Tagliabue, Antoniani, Maciag, Bocci, et al. 2010). Of note, absence of the DGC DosC correlates with *pga* presence in strains Fec67, B‐11870 and No.12; additionally, the *dosP* ORF contains a stop codon in B‐11870. Strain Tob1 lacks DosC, the *pga* operon and the DGC YcdT, whereas DosP is present. Strains Fec101 and B‐8638 contain both DosC and DosP, but lack the *pga* operon along with *ycdT*.

Besides YciR, we identified several c‐di‐GMP turnover proteins that could potentially contribute to the semi‐constitutive rdar morphotype of the strains. Experimental evidence for a variability of the c‐di‐GMP protein network has been gathered previously. Probiotic and commensal *E. coli* strains with production of cellulose independent of YedQ and AdrA had been observed leaving the DGC required for cellulose production to be identified (Da Re & Ghigo, 2006; Monteiro et al., 2009). On the other hand, PDE activity also affects *csgD* expression. Deletion of the *yjcC* homologue STM4264 in *S*. Typhimurium promoted temperature‐independent *csgD* expression (Simm et al., 2007). YjcC_Fec10_ is identical to the K‐12 reference sequence, whereas the YjcC proteins from the semi‐constitutive biofilm strains exhibit not only several aa substitutions, but also a C‐terminal truncation (Table S3). Whether these aa differences decrease YjcC functionality and thus promote temperature‐independent CsgD and CsgD‐independent cellulose expression (Figure 1a) needs to be shown in follow‐up studies.

Of note, YhjH has been identified as the key PDE to regulate motility (Le Guyon et al., 2015; Pesavento et al., 2008; Simm et al., 2007). Although YhjH was conserved in all 61 strains (Povolotsky & Hengge, 2016), one or two aa substitutions are present in YhjH of the semi‐constitutive rdar strains except for Fec101. It remains to be tested whether these aa substitutions lead to altered YhjH activity.

### 4.4 Evolution of c‐di‐GMP turnover proteins mediates variable modulation of biofilm formation

In the investigated strains, YciR is a major target of mutations that lead to altered protein activity. The nonsense mutation changing the TGG codon to a TAG stop codon in the urosepsis strain B‐11870 is reflected by a TGA stop codon at the same position in the EHEC strain O111:H‐ 11128 (Povolotsky & Hengge, 2016) indicating that a truncated YciR lacking the EAL domain is not unique to B‐11870.

In *E. coli*, YciR is proposed to inhibit the DGC YdaM and MlrA through protein–protein interactions in a c‐di‐GMP‐dependent manner, to downregulate *csgD* expression. YdaM and MlrA are then released upon rising c‐di‐GMP levels (Hengge, 2016; Lindenberg et al., 2013). A more pronounced effect of EAL domain mutants in downregulation of the rdar morphotype (Figure 4) apparently confirms the previously described unresponsiveness to c‐di‐GMP levels with tighter binding of YdaM and MlrA. Thus, rdar morphotype and *csgD* regulation is not dependent on the PDE activity of YciR, as reported for *E. coli* K‐12 (Hengge, 2016). Protein variants with mutations K581A and E617A had no effect suggesting impaired expression as observed for *S*. Typhimurium (Ahmad et al., 2017).

**Figure 5:**
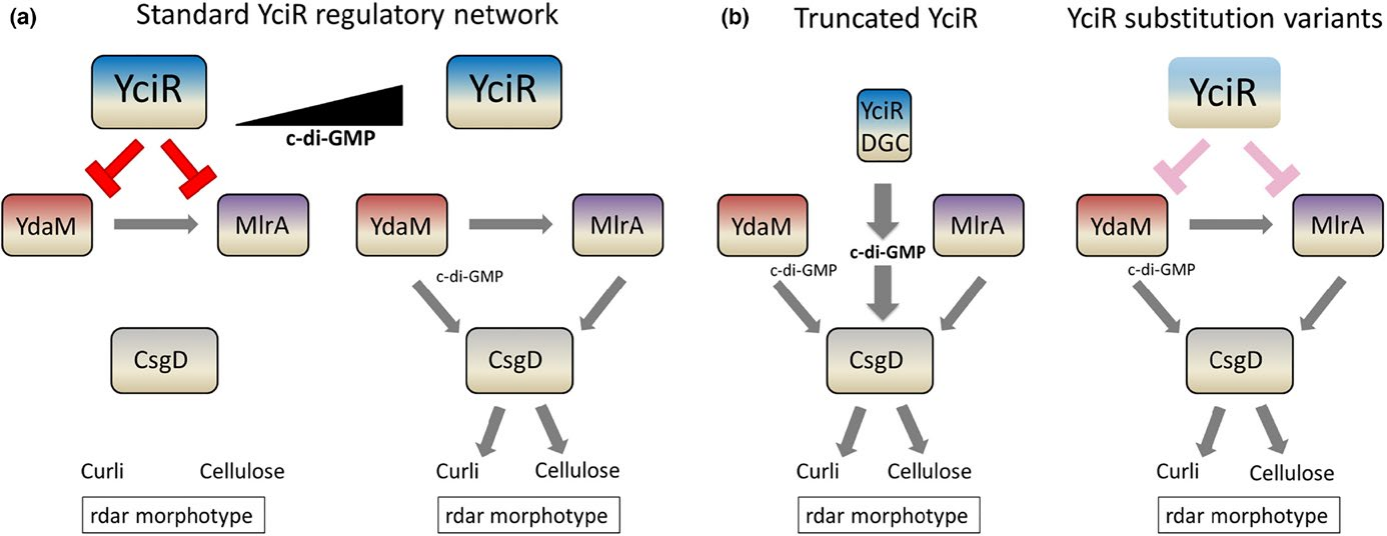
Means of YciR‐based regulation of *csgD* expression. (a) In *E. coli* K‐12 model strain, the trigger enzyme YciR can directly bind to and interfere with the functionality of DGC YdaM and MlrA, a transcriptional regulator activating *csgD* expression. Suppression is relieved upon high cyclic di‐GMP levels. (b) YciR‐mediated regulation can be adjusted by introduction of a stop codon within the ORF, leading to solely expression of the PAS‐PAC‐GGDEF domain (left) or by amino acid substitutions interfering with the expression/functionality of YciR (right)

In functionally restricted YciR_Tob1_, we observed aa mutations outside of the catalytic motifs throughout the protein sequence compared to the fully functional YciR_Fec10_ and YciR_Fec101_ proteins, which affects functionality and/or stability as observed by a reduced ability to downregulate the rdar morphotype. Two major determinative aa that contribute to YciR functionality and/or stability were identified at 28°C. At 37°C, all aa except four alter YciR functionality suggesting a different mechanism of action. The various modes of YciR action on the semi‐constitutive rdar morphotype and *csgD* expression are summarized in Figure 5.

Interestingly, overexpression of YciR_Fec10_, YciR_Fec101_, and YciR_Tob1_ resulted in the absence of a band of approximately 37 kDa from a denaturating SDS‐PAGE that was identified by mass spectrometry as the outer membrane protein OmpC (Nimtz, M et al., data not shown). Of note, OmpC was present upon overexpression of the truncated YciR_B‐11870_. Thus, the EAL domain of highly active and restricted YciR proteins is involved in downregulating OmpC. This finding suggests a first additional functional role of YciR beyond *csgD* expression that can be ascribed to the EAL domain of the protein.

*E. coli* isolates show a surprising diversity in c‐di‐GMP turnover proteins. Thus, the c‐di‐GMP signaling network and consequently regulation of biofilm formation is highly plastic. Indeed, individual isolates have evolved different modes to alter rdar biofilm formation by modulation of YciR function. Already single aa substitutions alter the functionality of the trigger enzyme YciR substantially. Previously, single aa substitutions in DGCs have been shown to dramatically affect rugose colony morphology biofilms (Beyhan & Yildiz, 2007). In this context, it should be mentioned that the choice of codons can affect translation efficiency and protein function (Behera, Kutty, Kumar, & Sharma, 2016). Besides YciR, other c‐di‐GMP turnover proteins showed a high degree of aa substitutions which can additively contribute to the semi‐constitutive rdar morphotype at 28°C and 37°C.

Moreover, the original, reverse, functionality of the GGDEF‐EAL domain protein YciR can be recovered by conversion of a nonsense into a sense codon as shown for YciR_B‐11870_. However, YciR is not the only c‐di‐GMP turnover protein with disrupted or shortened open reading frame. This work shows that single aa substitutions can fine‐tune protein activity of signaling networks more than previously appreciated which occur as adaptation to changing environmental or host conditions. Our results indicate increasing rearrangement of the c‐di‐GMP network from phylotype A (K‐12, Fec10) over B1 (Fec101) to phylotype B2 strains (Tob1, Fec67, No. 12, B‐11870). What exactly drives the evolution of c‐di‐GMP turnover proteins in the different phylotypes is a question for future studies.

## ORIGINALITY‐SIGNIFICANCE STATEMENT

This study is the first to address the molecular mechanisms behind semi‐constitutive rdar biofilm expression of both commensal and uropathogenic *E. coli* isolates. We employed genome sequencing to directly link genome alterations to distinct, semi‐constitutive, rdar biofilm phenotypes. This work significantly contributes to our understanding of the variability and development of the cyclic di‐GMP network in *E. coli* and highlights the impact of single amino acid mutations for protein functionality.

## CONFLICT OF INTEREST

None declared.
