# Supplementary material for: Alterations of c‐di‐GMP turnover proteins modulate semi‐constitutive rdar biofilm formation in commensal and uropathogenic Escherichia coli
Source: Microbiologyopen. 2017 Sep 15;6(5):e00508. doi: 10.1002/mbo3.508 (PMC5635171; doi:10.1002/mbo3.508)
Supplement: Supplementary file 1 [file MBO3-6-na-s001.pdf]

Supplementary data

Cimdins A, Simm R, Li F, et al.
Alterations of c-di-GMP turnover proteins modulate semi-constitutive rdar biofilm formation in commensal and uropathogenic *Escherichia coli*. *MicrobiologyOpen*. 2017;e508.
https://doi.org/10.1002/mbo3.508

Additional files

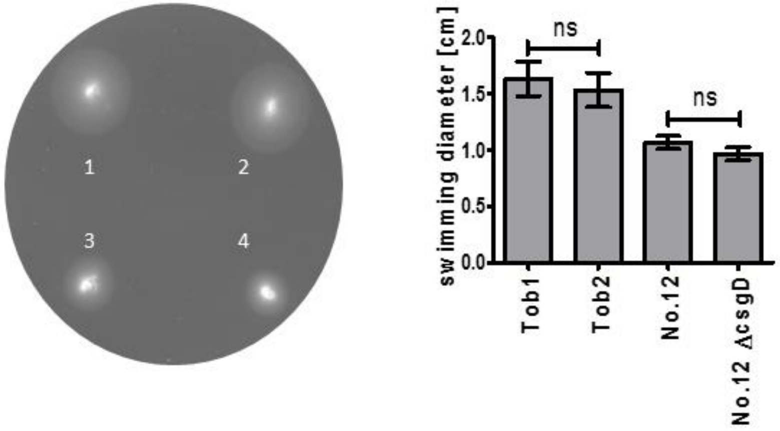

Fig. S1 Swimming motility is independent of CsgD.

Swimming motility is not dependent on *csgD*. Motility of the strains Tob1 and No.12 was indistinguishable from the respective *csgD* mutants (Tob2 (Tob1 Δ*csgD*) and No.12 Δ*csgD*). 1= Tob1, 2= Tob2, 3= No.12, 4= No.12 Δ*csgD*.

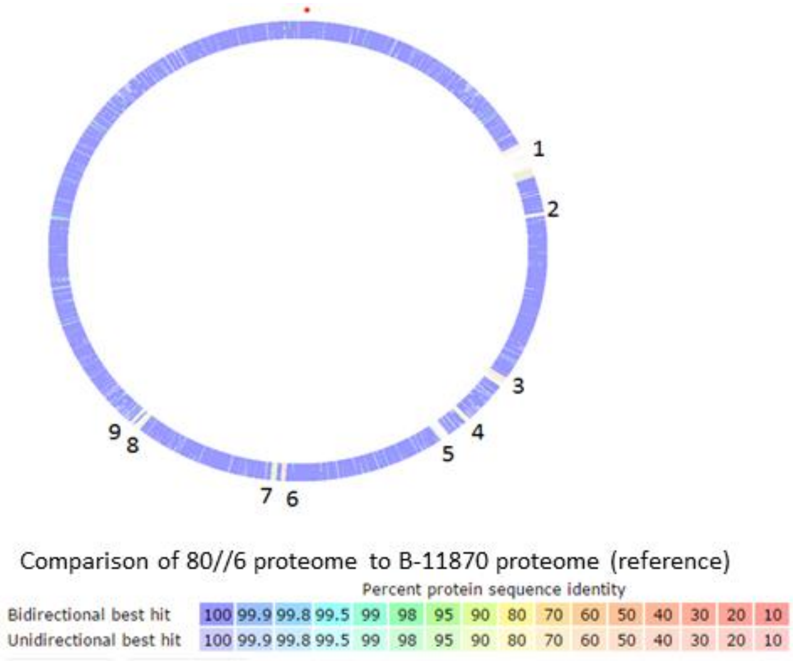

Fig. S2 RAST proteome comparison of 80//6 and B-11870.

Comparison of the whole proteome between clonal variants B-11870 and 80//6 using the RAST server. The B-11870 genome contains additional regions not present in 80//6. These regions correspond to insertions of foreign DNA elements with a total number of 241 hits for genes present in B-11870 but not in 80//6, at 9 major locations in the chromosome.

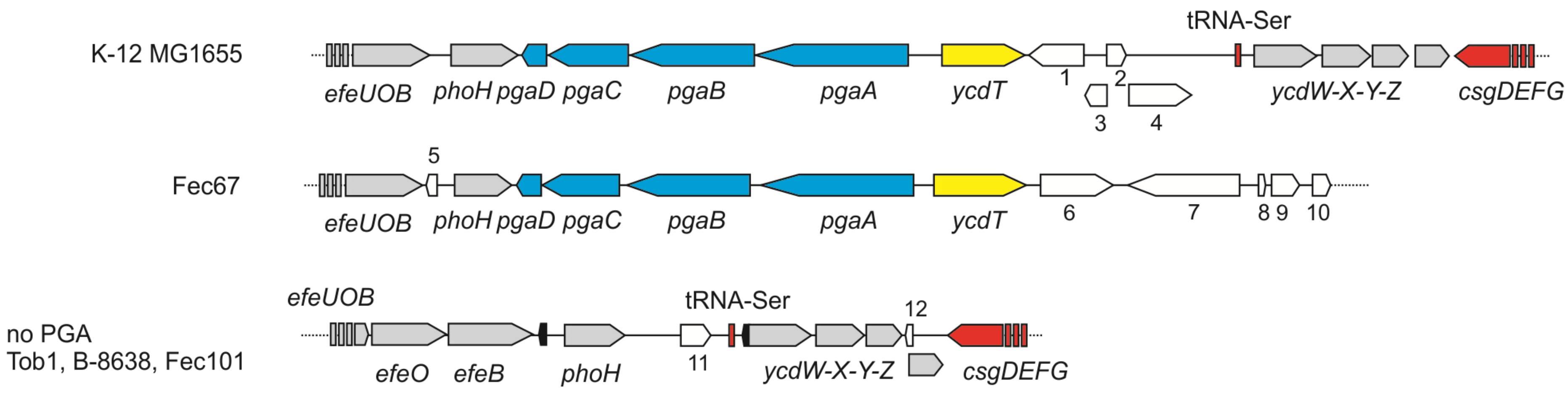

Fig. S3: Genomic context of the *ycdT-pga* locus.

Visualization of the *ycdT-pga* flanking regions in *E. coli* K-12 MG1655 (top row) and Fec67, and the corresponding region in the *pga* negative strains Tob1, Fec101, and B-8638 as annotated by RAST server. Genes of interest are displayed in colour. Grey coloring indicates similar genomic location, while white coloured genes are uniquely positioned. Black labelled genes are ORFs encoding hypothetical proteins only predicted for B-8638, but not for Tob1 or Fec101.

1 *insF1*, **2** *insE1*, **3** *ymdE*, **4** *ycdU*, **5** hypothetical protein, **6** integrase, **7** putative membrane protein, **8** hypothetical protein, **9** mobile element, **10** *ybaK*, **11** hypothetical protein, **12** hypothetical protein

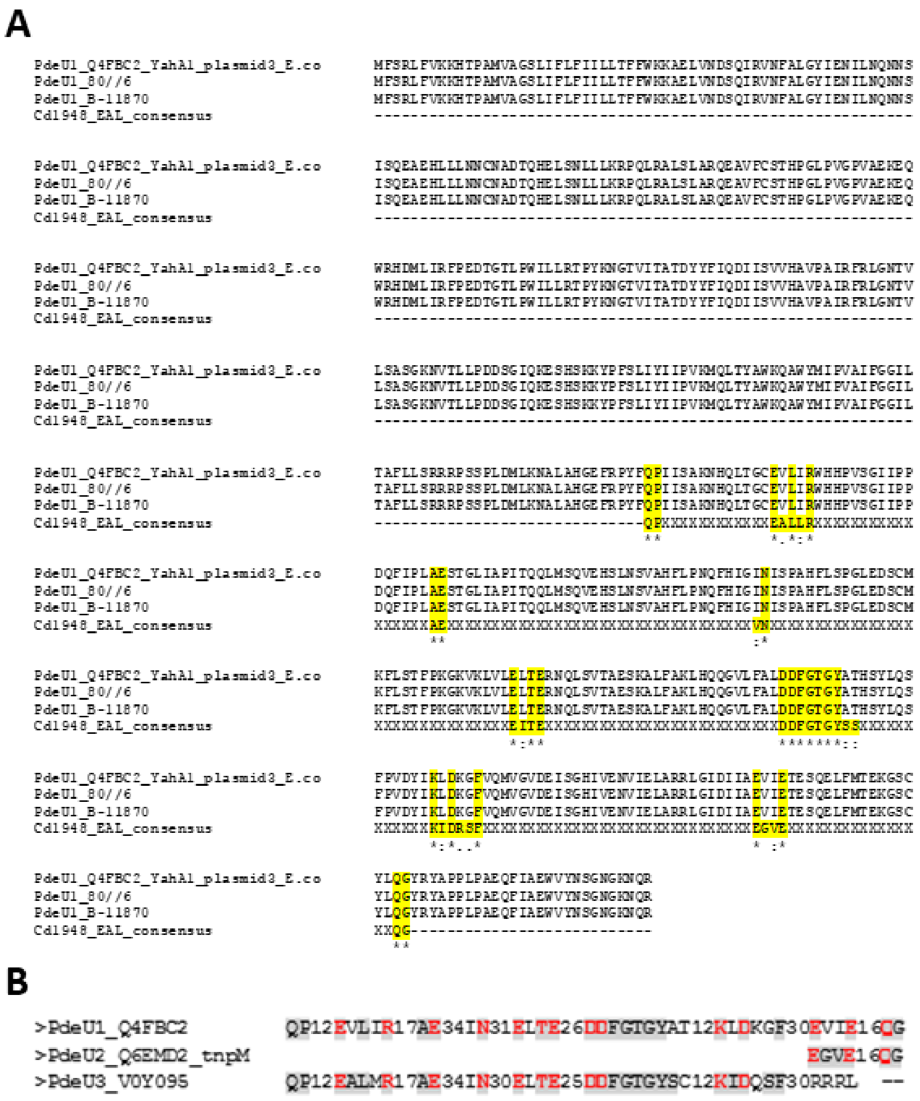
A
PdeU1_Q4FBC2_YahA1_plasmid3_E.co MFSRLFVKKHTPAMVAGSLIFLFIILLTFFWKKAELVNDSQIRVNFALGYIENILNQNNS
PdeU1_80//6 MFSRLFVKKHTPAMVAGSLIFLFIILLTFFWKKAELVNDSQIRVNFALGYIENILNQNNS
PdeU1_B-11870 MFSRLFVKKHTPAMVAGSLIFLFIILLTFFWKKAELVNDSQIRVNFALGYIENILNQNNS
Cd1948_EAL_consensus --
PdeU1_Q4FBC2_YahA1_plasmid3_E.co ISQEAEHLLLNNCNADTQHELSNLLLKRPQLRALSLARQEAVFCSTHPGLPVGPVAEKEQ
PdeU1_80//6 ISQEAEHLLLNNCNADTQHELSNLLLKRPQLRALSLARQEAVFCSTHPGLPVGPVAEKEQ
PdeU1_B-11870 ISQEAEHLLLNNCNADTQHELSNLLLKRPQLRALSLARQEAVFCSTHPGLPVGPVAEKEQ
Cd1948_EAL_consensus --
PdeU1_Q4FBC2_YahA1_plasmid3_E.co WRHDMLIRFPEDTGTLPWILLRTPYKNGTVITATDYYFIQDIISVVHAVPAIRFRLGNTV
PdeU1_80//6 WRHDMLIRFPEDTGTLPWILLRTPYKNGTVITATDYYFIQDIISVVHAVPAIRFRLGNTV
PdeU1_B-11870 WRHDMLIRFPEDTGTLPWILLRTPYKNGTVITATDYYFIQDIISVVHAVPAIRFRLGNTV
Cd1948_EAL_consensus --
PdeU1_Q4FBC2_YahA1_plasmid3_E.co LSASGKNVTLLPDDSGIQKESHSKKYPFSLIYIIPVKMQLTYAWKQAWYMIPVAIFGGIL
PdeU1_80//6 LSASGKNVTLLPDDSGIQKESHSKKYPFSLIYIIPVKMQLTYAWKQAWYMIPVAIFGGIL
PdeU1_B-11870 LSASGKNVTLLPDDSGIQKESHSKKYPFSLIYIIPVKMQLTYAWKQAWYMIPVAIFGGIL
Cd1948_EAL_consensus --
PdeU1_Q4FBC2_YahA1_plasmid3_E.co TAFLLSRRRPSSPLDMLKNALAHGEFRPYFQPIISAKNHQLTGCEVLIRWHHPVSGIIPP
PdeU1_80//6 TAFLLSRRRPSSPLDMLKNALAHGEFRPYFQPIISAKNHQLTGCEVLIRWHHPVSGIIPP
PdeU1_B-11870 TAFLLSRRRPSSPLDMLKNALAHGEFRPYFQPIISAKNHQLTGCEVLIRWHHPVSGIIPP
Cd1948_EAL_consensus -----------------------------QPXXXXXXXXXXXEALLRXXXXXXXXXX
PdeU1_Q4FBC2_YahA1_plasmid3_E.co DQFIPLAESTGLIAPITQQLMSQVEHSLNSVAHFLPNQFHIGINISPAHFLSPGLEDSCM
PdeU1_80//6 DQFIPLAESTGLIAPITQQLMSQVEHSLNSVAHFLPNQFHIGINISPAHFLSPGLEDSCM
PdeU1_B-11870 DQFIPLAESTGLIAPITQQLMSQVEHSLNSVAHFLPNQFHIGINISPAHFLSPGLEDSCM
Cd1948_EAL_consensus XXXXXXAEXXXXXXXXXXXXXXXXXXXXXXXXXXXXXXXXXXVNXXXXXXXXXXXXXX
PdeU1_Q4FBC2_YahA1_plasmid3_E.co KFLSTFPKGKVKLVLELTERNQLSVTAESKALFAKLHQQGVLFALDDFGTGYATHSYLQS
PdeU1_80//6 KFLSTFPKGKVKLVLELTERNQLSVTAESKALFAKLHQQGVLFALDDFGTGYATHSYLQS
PdeU1_B-11870 KFLSTFPKGKVKLVLELTERNQLSVTAESKALFAKLHQQGVLFALDDFGTGYATHSYLQS
Cd1948_EAL_consensus XXXXXXXXXXXXXXEITEXXXXXXXXXXXXXXXXXXXXXXXXDDFGTGYSSXXXXXX
PdeU1_Q4FBC2_YahA1_plasmid3_E.co FPVDYIKLDKGFVQMVGVDEISGHIVENVIELARRLGIDIIAEVIETESQELFMTEKGSC
PdeU1_80//6 FPVDYIKLDKGFVQMVGVDEISGHIVENVIELARRLGIDIIAEVIETESQELFMTEKGSC
PdeU1_B-11870 FPVDYIKLDKGFVQMVGVDEISGHIVENVIELARRLGIDIIAEVIETESQELFMTEKGSC
Cd1948_EAL_consensus XXXXXXKIDRSFXXXXXXXXXXXXXXXXXXXXXXXXXXXXXEGVEXXXXXXXXXXXXX
PdeU1_Q4FBC2_YahA1_plasmid3_E.co YLQGYRYAPPLPAEQFIAEWVYNSGNGKNQR
PdeU1_80//6 YLQGYRYAPPLPAEQFIAEWVYNSGNGKNQR
PdeU1_B-11870 YLQGYRYAPPLPAEQFIAEWVYNSGNGKNQR
Cd1948_EAL_consensus XXQG---------------------------
B
>PdeU1_Q4FBC2 QP12EVLIR17AE34IN31ELTE26DDFGTGYAT12KLDKGF30EVIE16QG
>PdeU2_Q6EMD2_tnpM EGVE16QG
>PdeU3_V0Y095 QP12EALMR17AE34IN30ELTE25DDFGTGYSC12KIDQSF30RRRL --

Fig. S4: Multiple alignment of novel phosphodiesterases identified in the investigated strains.

A Sequence comparison of PdeU1 from 80//6 and B-11870 to a PdeU1 reference sequence annotated for *E. coli* (Uniprot entry: Q4FBC2_ECOLX) and the PFAM consensus motifs for an active EAL domain. MUSCLE (http://www.ebi.ac.uk/Tools/msa/muscle/) was used to create the alignment. Aa identical to the consensus motif are on a yellow background. **B** Comparison of conserved EAL domain catalytic motifs in PdeU1 (Uniprot entry: Q4FBC2_ECOLX), PdeU2 (Uniprot entry: Q6EMD2_ECOLX), and PdeU3 (Uniprot entry: V0Y095_ECOLX).

A

```
YciR_Tob1         MKTVRESTTLYNFLGSHNPYWRLTESSDVLRFSTTEITEPDRILQLSAEQAARIREMTVI
YciR_B-11870      MKTVRESTTLYNFLGSHNPYWRLTESSDVLRFSTTEITEPDRILQLSAEQAARIREMTVI
YciR_Fec101       MKTVRESTTLYNFLGSHNPYWRLTESSDVLRFSTTETTEPDRTLQLSAEQAARIREMTVI
YciR_Fec10        MKTVRESTTLYNFLGSHNPYWRLTESSDVLRFSTTETTEPDRTLQLSAEQAARIREMTVI
                  ************************************ ***** *****************

YciR_Tob1         TSSLMMSLTVDESDLSVHLVGRKINKREWGGNASAWHDTPAVARDLSHGLSFAEQVVSEA
YciR_B-11870      TSSLMMSLTVDESDLSVHLVGRKINKREWGGNASAWHDTPAVARDLSHGLSFAEQVVSEA
YciR_Fec101       TSSLMMSLTVDESDLSVHLVGRKINKREWAGNASAWHDTPAVARDLSHGLSFAEQVVSEA
YciR_Fec10        TSSLMMSLTVDESDLSVHLVGRKINKREWAGNASAWHDTPAVARDLSHGLSFAEQVVSEA
                  *****************************.******************************

YciR_Tob1         HSAIVILDSRGNIQRFNRLCEDYTGLKEHDVIGQSMFKLFMSRREAAASRRNNRVFFRSG
YciR_B-11870      HSAIVILDSRGNIQRFNRLCEDYTGLKEHDVIGQSVFKLFMSRREAAASRRNNRVFFRSG
YciR_Fec101       HSAIVILDSRGNIQRFNRLCEDYTGLKEHDVIGQSVFKLFMSRREAAASRRNNRVFFRSG
YciR_Fec10        HSAIVILDSRGNIQRFNRLCEDYTGLKEHDVIGQSVFKLFMSRREAAASRRNNRVFFRSG
                  ***********************************:************************

YciR_Tob1         NAYEVELWIPTRKGQRLFLFRNKFVHSGSGKNEIFLICSGTDITEERRAQERLRILANTD
YciR_B-11870      NAYEVELWIPTRKGQRLFLFRNKFVHSGSGKNEIFLICSGTDITEERRAQERLRILANTD
YciR_Fec101       NAYEVELWIPTRKGQRLFLFRNKFVHSGSGKNEIFLICSGTDITEERRAQERLRILANTD
YciR_Fec10        NAYEVELWIPTCKGQRLFLFRNKFVHSGSGKNEIFLICSGTDITEERRAQERLRILANTD
                  *********** ************************************************

YciR_Tob1         SITGLPNRNAMQELIDHAINQADNNKVGVVYLDLDNFKKVNDAYGHLFGDQLLRDVSLAI
YciR_B-11870      SITGLPNRNAMQELIDHAINQADNNKVGVVYLDLDNFKKVNDAYGHLFGDQLLRDVSLAI
YciR_Fec101       SITGLPNRNAMQDLIDHAINHADNNKVGVVYLDLDNFKKVNDAYGHLFGDQLLRDVSLAI
YciR_Fec10        SITGLPNRNAMQDLIDHAINHADNNKVGVVYLDLDNFKKVNDAYGHLFGDQLLRDVSLAI
                  ************:*******:***************************************

YciR_Tob1         LSCLEHDQVLARPGGDEFLVLASNTSQSALEAMASRILTRLRLPFRIGLIEVYTSCSVGI
YciR_B-11870      LSCLEHDQVLARPGGDEFLVLASNTSQSALEALASRILTRLRLPFRIGLIEVYTSCSVGI
YciR_Fec101       LSCLEHDQVLARPGGDEFLVLASNTSQSALEAMASRILTRLRLPFRIGLIEVYTSCSVGI
YciR_Fec10        LSCLEHDQVLARPGGDEFLVLASNTSQSALEAMASRILTRLRLPFRIGLIEVYTSCSVGI
                  ********************************:***************************

YciR_Tob1         SLSPEHGSDSAAIIRHADTAMYTAKEGGRGQFCVFTPEMNQRVFEYLWLDTNLRKALEND
YciR_B-11870      SLSPEHGSDSAAIIRHADTAMYTAKEGGRGQFCVFTPEMNQRVFEYLWLDTNLRKALEND
YciR_Fec101       ALSPEHGSDSTAIIRHADTAMYTAKEGGRGQFCVFTPEMNQRVFEYLWLDTNLRKALEND
YciR_Fec10        ALSPEHGSDSTAIIRHADTAMYTAKEGGRGQFCVFTPEMNQRVFEYLWLDTNLRKALEND
                  :*********:*************************************************

YciR_Tob1         QLVIHYQPKITWRGEVRSLEALVRWQSPERGLIPPLDFISYAEESGLIVPLGRWVILDVV
YciR_B-11870      QLVIHYQPKIT-RGEVRSLEALVRWQSPERGLIPPLDFISYAEESGLIVPLGRWVILDVV
YciR_Fec101       QLVIHYQPKITWRGEVRSLEALVRWQSPERGLIPPLDFISYAEESGLIVPLGRWVILDVV
YciR_Fec10        QLVIHYQPKITWRGEVRSLEALVRWQSPERGLIPPLDFISYAEESGLIVPLGRWVILDVV
                  *********** ************************************************

YciR_Tob1         RQVAKWRDKGINLRVAVNISARQLADQTIFTALKQVLQELNFEYCPIDVELTESCLIEND
YciR_B-11870      RQVAKWRDKGINLRVAVNISARQLADQTIFTALKQVLQELNFEYCPIDVELTESCLIEND
YciR_Fec101       RQVAKWRDKGINLRVAVNISARQLADQTIFTALKQVLQELNFEYCPIDVELTESCLIEND
YciR_Fec10        RQVAKWRDKGINLRVAVNISARQLADQTIFTALKQVLQELNFEYCPIDVELTESCLIEND
                  ************************************************************

YciR_Tob1         ELALSVIQQFSRLGAQVHLDDFGTGYSSLSQLARFPIDAIKLDQVFVRDIHKQPVSQSLV
YciR_B-11870      ELALSVIQQFSRLGAQVHLDDFGTGYSSLSQLARFPIDAIKLDQVFVRDIHKQPVSQSLV
YciR_Fec101       ELALSVIQQFSQLGAQVHLDDFGTGYSSLSQLARFPIDAIKLDQVFVRDIHKQPVSQSLV
YciR_Fec10        ELALSVIQQFSQLGAQVHLDDFGTGYSSLSQLARFPIDAIKLDQVFVRDIHKQPVSQSLV
                  ***********.************************************************

YciR_Tob1         RAIVAVAQALNLQVIAEGVESAKEDAFLTKNGINERQGFLFAKPMPAVAFERWYKRYLKR
YciR_B-11870      RAIVAVAQALNLQVIAEGVESAKEDAFLTKNGINERQGFLFAKPMPAVAFERWYKRYLKR
YciR_Fec101       RAIVAVAQALNLQVIAEGVESAKEDAFLTKNGINERQGFLFAKPMPAVAFERWYKRYLKR
YciR_Fec10        RAIVAVAQALNLQVIAEGVESAKEDAFLTKNGINERQGFLFAKPMPAVAFERWYKRYLKR
                  ************************************************************

YciR_Tob1         T
YciR_B-11870      T
YciR_Fec101       A
YciR_Fec10        A
                  :
```

B

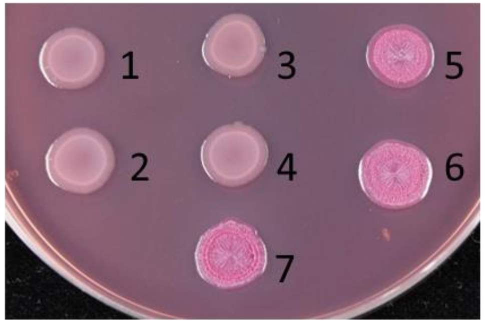

C

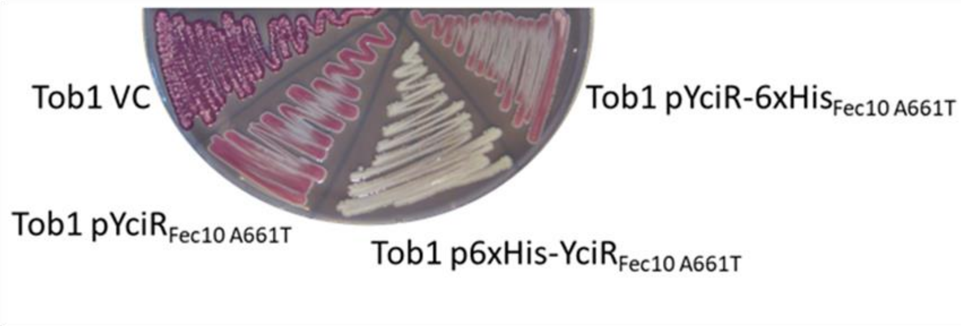

Fig. S5 Effect of YciR overexpression on rdar morphotype is dependent on the position of the 6x-His tag.

A Amino acid comparison of YciR from Fec10, Tob1, Fec101 and B-11870 performed with MUSCLE. Non-conserved aa are indicated on a grey background. B-11870 is truncated, but upon reversion of the stop codon (TAG) to the aa tryptophane (TGG), B-11870_Full is restored. **B** The activity of YciR on rdar morphotype formation is not affected upon substitution of the final amino acid alanine to threonine and vice versa: YciR variants cloned in pBAD30 were expressed in strain Tob1 and colony morphology on CR-plates was assessed after growth at 37°C for 24h with 0.1% L-arabinose as inducer. Strain Tob1 with: 1= pYciR$_{\text{Fec101}}$ (wild type); 2= pYciR$_{\text{Fec101 A661T}}$ (designated as wild type in this work); 3= pYciR$_{\text{Fec10}}$ (wild type); 4= pYciR$_{\text{Fec10 A661T}}$ (designated as wild type in this work); 5= pYciR$_{\text{Tob1 T661A}}$; 6= pYciR$_{\text{Tob1}}$ (wild type); 7= VC. pYciR=YciR cloned in pBAD30. VC=pBAD30 **C** Rdar morphotype formation of strain Tob1 upon overexpression of YciR$_{\text{Fec10 A661T}}$ (designated as wild type in this work) with no tag, N-terminal 6xHis-tag, and C-terminal 6xHis-tag. YciR$_{\text{Fec10 A661T}}$ with N-terminal 6xHis-tag downregulated rdar morphotype formation most effectively, while YciR$_{\text{Fec10 A661T}}$ with a C-terminal 6xHis-tag still showed increased ability to downregulate rdar morphotype expression compared to untagged YciR$_{\text{Fec10 A661T,}}$ but leads to a colony colour change. Rdar colony morphology on CR-plates was assessed after growth at 28°C for 48h with 0.1% L-arabinose as inducer. VC= pBAD30. pYciR=YciR cloned in pBAD30.

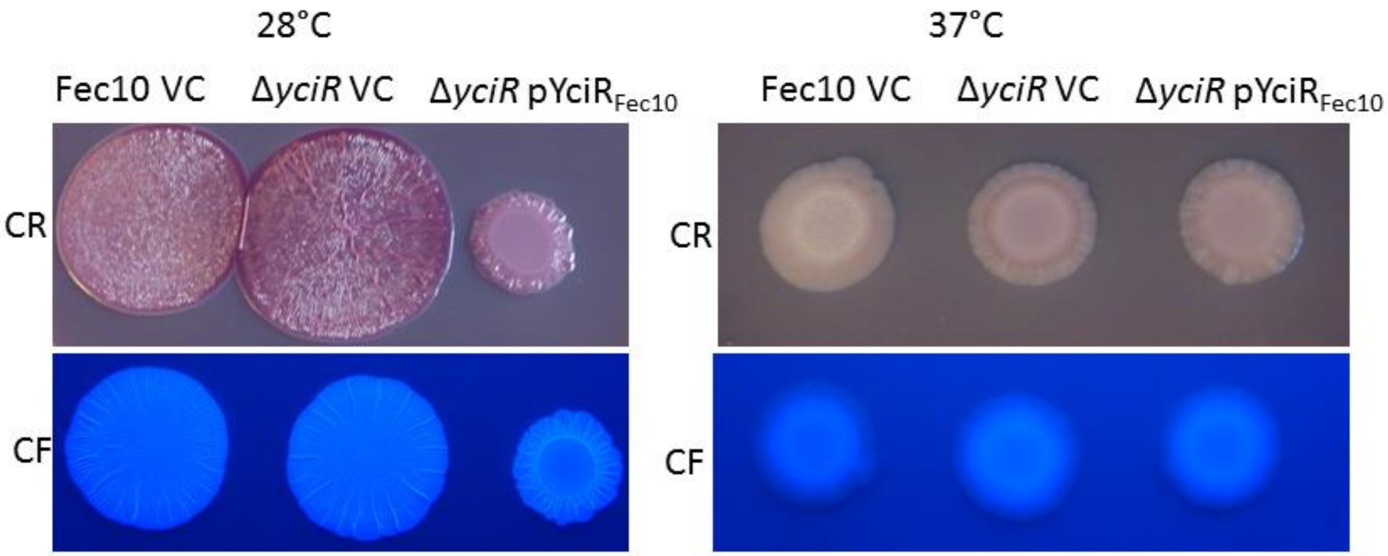

Fig. S6 YciR deletion results in a moderately upregulated rdar morphotype in the commensal strain Fec10.

Rdar colony morphology of Fec10 upon deletion of *yciR*. Analysis of rdar morphotype of Fec10 VC, Fec10 Δ*yciR* VC and Fec10 Δ*yciR* pYciR$_{Fec10}$ (pBAD30::YciR$_{Fec10\ A661T}$). Colony morphology was investigated after 48 h at 28°C and 37°C. LB without salt agar plates were supplemented with 0.1% Arabinose, Congo Red (CR; upper row) or Calcofluor (CF, lower row). Note that overexpression of YciR$_{Fec10}$ leads to hyper complementation of the rdar colony morphotype. VC= pBAD30

Table S1: Plasmids used in the study

**Plasmid**	**Alternative name**	**Relevant characteristics**	**Reference**
pBAD30		Cloning vector, pACYC origin, arabinose inducible pBAD promoter, Amp^{r}	(Guzman et al., 1995)
pKD3		Template plasmid for λ red mediated recombination, Cm^{r}, Amp^{r}	(Datsenko and Wanner, 2000)
pKD46		pBAD promoter, encodes λ red recombinase, Amp^{r}	(Datsenko and Wanner, 2000)
pBAD30::6xHis-YciR$_{\text{Fec10 A661T}}$	pYciR5	Amp^{r}, YciR with N-terminal 6xHis tag	This study
pBAD30::YciR-6xHis$_{\text{Fec10 A661T}}$	pYciR6	Amp^{r}, YciR with C-terminal 6xHis tag	This study
pBAD30::YciR$_{\text{Fec10 A661T}}$	pYciR39	Amp^{r}, YciR without tag	This study
pBAD30::YciR$_{\text{Fec10}}$	pYciR40	Based on pYciR5	This study
pBAD30::YciR$_{\text{Fec10 T37I}}$	pYciR25	Based on pYciR5	This study
pBAD30::YciR$_{\text{Fec10 T43I}}$	pYciR26	Based on pYciR5	This study
pBAD30::YciR$_{\text{Fec10 A90G}}$	pYciR27	Based on pYciR5	This study
pBAD30::YciR$_{\text{Fec10 V156M}}$	pYciR28	Based on pYciR5	This study
pBAD30::YciR$_{\text{Fec10 D253E}}$	pYciR29	Based on pYciR5	This study
pBAD30::YciR$_{\text{Fec10 H261Q}}$	pYciR30	Based on pYciR5	This study

pBAD30::YciR$_{Fec10\ A361S}$	pYciR31	Based on pYciR5	This study
pBAD30::YciR$_{Fec10\ T371A}$	pYciR32	Based on pYciR5	This study
pBAD30::YciR$_{Fec10\ Q552R}$	pYciR33	Based on pYciR5	This study
pBAD30::YciR$_{Fec10\ T37I,\ T371A}$	pYciR34	Based on pYciR25	This study
pBAD30::YciR$_{Fec101\ A661T}$	pYciR7	Amp^r, YciR with N-terminal 6xHis tag	This study
pBAD30::YciR$_{Fec101}$	pYciR41	Based on pYciR 7	This study
pBAD30::YciR$_{Fec101\ E317A}$	pYciR18	Based on pYciR7, GGDAF mutation	This study
pBAD30::YciR$_{Fec101\ D316A\ E317A}$	pYciR11	Based on pYciR7, GGAAF mutation	This study
pBAD30::YciR$_{Fec101\ E440A}$	pYciR12	Based on pYciR7, AAL mutation	This study
pBAD30::YciR$_{Fec101\ E317A\ E440A}$	pYciR17	Based on pYciR12, GGDAF-AAL mutation	This study
pBAD30::YciR$_{Fec101\ D316A\ E317A\ E440A}$	pYciR16	Based on pYciR12, GGAAF-AAL mutation	This study
pBAD30::YciR$_{Fec101\ E617A}$	pYciR15	Based on pYciR7, AGVE mutation	This study
pBAD30::YciR$_{Fec101\ E530A}$	pYciR13	Based on pYciR7, ALTE mutation	This study
pBAD30::YciR$_{Fec101\ K581A}$	pYciR14	Based on pYciR7	This study
pBAD30::YciR$_{Fec101\ T37I}$	pYciR35	Based on pYciR7	This study
pBAD30::YciR$_{Fec101\ T371A}$	pYciR36	Based on pYciR7	This study
pBAD30::YciR$_{Fec101\ T37I\ V156M}$	pYciR37	Based on pYciR35	This study
pBAD30::YciR$_{Fec101\ T37I\ A361S}$	pYciR38	Based on pYciR35	This study

pBAD30::YciR$_{\text{Tob1}}$	pYciR3	Amp^{r}, YciR with N-terminal 6xHis tag	This study
pBAD30::YciR$_{\text{Tob1 T661A}}$	pYciR42	Based on pYciR3	This study
pBAD30::YciR$_{\text{Tob1 E317A}}$	pYciR23	Based on pYciR3, GGDAF mutation	This study
pBAD30::YciR$_{\text{Tob1 D316A E317A}}$	pYciR19	Based on pYciR3, GGAAF mutation	This study
pBAD30::YciR$_{\text{Tob1 E440A}}$	pYciR20	Based on pYciR3, AAL mutation	This study
pBAD30::YciR$_{\text{Tob1 E317A E440A}}$	pYciR24	Based on pYciR20, GGDAF-AAL mutation	This study
pBAD30::YciR$_{\text{Tob1 K581A}}$	pYciR21	Based on pYciR3	This study
pBAD30::YciR$_{\text{B-11870}}$	pYciR1	Amp^{r}, YciR with N-terminal 6xHis tag	This study
pBAD30::YciR$_{\text{B-11870_Full}}$	pYciR9	Amp^{r}, YciR with N-terminal 6xHis tag,<br>stop codon revertant	This study

Datsenko, K.A., and Wanner, B.L. (2000). One-step inactivation of chromosomal genes in *Escherichia coli* K-12 using PCR products. Proc Natl Acad Sci U S A *97*, 6640-6645.

Guzman, L.M., Belin, D., Carson, M.J., and Beckwith, J. (1995). Tight regulation, modulation, and high-level expression by vectors containing the arabinose PBAD promoter. J Bacteriol *177*, 4121-4130.

Table S2: Oligonucleotides used in the study

Name	Sequence	Use of oligonucleotide primer
yciR-Nhis-Start	CTATG*TCTAGA*ATG**CACCATCACCACCATCAT**AAAACCGTTAGGGAGTCCAC	Cloning YciR of B-11870, Tob1, Fec101 A661T, Fec10 A661T with N-terminal His-tag
yciR_Fec10	CTATG*TCTAGA*ATGAAAACCGTTAGGGAGTCCAC	Cloning of YciR$_{Fec10\ A661T}$ without N-terminal His-tag
yciR-Nhis-Stop	AATTGA*AAGCTT*TTATGTGCGCTTCAGGTAGCGTTTA	Cloning YciR of B-11870, Tob1, Fec101 A661T, Fec10 A661T
Fec10-wt-3'-for	ATCTGAAGCGC**G**CATAAAAGCTTG	Site directed mutagenesis of pYciR3, pYciR5 and pYciR7 to create YciR$_{TOB1(T661A)}$, YciR$_{FEC10}$ and YciR$_{Fec101}$
Fec10-wt-3'-rev	GCGCGCTTCAGATAGCGTTTATAC	Site directed mutagenesis of pYciR3, pYciR5 and pYciR7 to create YciR$_{TOB1(T661A)}$, YciR$_{FEC10}$ and YciR$_{Fec101}$
YciR-Stop_mut-for	CAACCGAAAATCACCT**G**GCGTGGCGAAGTGCG	Site-directed mutagenesis to revert stop codon in pYciR1
YciR-Stop_mut-rev	CGCACTTCGCCACGC**C**AGGTGATTTTCGGTTG	Site-directed mutagenesis to revert stop codon in pYciR1
yciR-FEC10-KO-For	ATGAAAACCGTTAGGGAGTCCACAACGTTGTACAACTTTCGTGTAGGCTGGAGCTGCTTC	Primers to create Δ*yciR* in Fec10
yciR-FEC10-KO-Rev	TTATGCGCGCTTCAGATAGCGTTTATACCAGCGTTCGAAGCATATGAATATCCTCCTTAG	Primer to create Δ*yciR* in strain Fec10
YciRTob1delfw	ATGAAAACCGTTAGGGAGTCCACAACGTTGTACAACTTTCGTGTAGGCTGGAGCTGCTTC	Primer to create Δ*yciR* in strain Tob1

YciRTob1delrv	TTATGTGCGCTTCAGGTAGCGTTTATA CCAGCGTTCGAAGCATATGAATATCCT CCTTAGT	Primer to create Δ*yciR* in strain Tob1
YciR-ctrl-fw	CAATGCGTTTAGCCGCCGTTAATTTC	Control primer for Δ*yciR* in strain Fec10 and Tob1
YciR-ctrl-rv	CAATATCAAT ACGTTCGGCA ACGCGC	Control primer for Δ*yciR* in strain Fec10 and Tob1
csgD_start	ATGTTTAATGAAGTCCATAGTATTCATG GTCATACATTATTGTTGATCACGTGTAG GCTGGAGCTGCTTC	Primer to create a *csgD* deletion in strain No.12
csgD_stop	TTATCGCCTGAGGTTATCGTTTGCCCA GGAAACCGCTTGTGTCCGGTTTTCATA TGAATATCCTCCTTAGT	Primer to create Δ*csgD* in strain No.12
csgD_forw2	CGATGAGTAAGGAGGGCTGA	Control primer for Δ*csgD* in strain No.12
csgD_rev2	CCTCATATCAACGGCGTTTT	Control primers for Δ*csgD* in strain No.12
ycir-T37I-fw	GCTTTTCTACCACCGAAA**T**CACAGAAC CTGATCGTA	Primer for site-directed mutagenesis on pYciR5 (pBAD30::YciR$_{Fec10\ A661T}$)
ycir-T37I-rv	TACGATCAGGTTCTGTG**A**TTTCGGTGG TAGAAAAGC	Primer for site-directed mutagenesis on pYciR5 (pBAD30::YciR$_{Fec10\ A661T}$)
YciR-T43I-fw	GTTCGGCAGATAACTGCAA**AA**TACGAT CAGGTTCTGTGGTT	Primer for site-directed mutagenesis on pYciR5 (pBAD30::YciR$_{Fec10\ A661T}$)
YciR-T43I-rv	AACCACAGAACCTGATCGTA**TT**TTGCA GTTATCTGCCGAAC	Primer for site-directed mutagenesis on pYciR5 (pBAD30::YciR$_{Fec10\ A661T}$)
ycir-A90G-fw	CAATAAACGGGAATGGG**GA**GGCAACG CGTCTGCATG	Primer for site-directed mutagenesis on pYciR5 (pBAD30::YciR$_{Fec10\ A661T}$)
ycir-A90G-rv	CATGCAGACGCGTTGCC**TC**CCCATTCC CGTTTATTG	Primer for site-directed mutagenesis on pYciR5 (pBAD30::YciR$_{Fec10\ A661T}$)

YciR_Fec10_V156Mfw	GACGTCATTGGGCAAAGC**A**TGTTTAAACTGTTTATGAGC	Primer for site-directed mutagenesis on pYciR5 (pBAD30::YciR$_{Fec10\ A661T}$) and on pYciR35 (pBAD30::YciR$_{Fec101T37I}$)
YciR_Fec10_V156Mrv	GCTCATAAACAGTTTAAACA**T**GCTTTGCCCAATGACGTC	Primer for site-directed mutagenesis on pYciR5 (pBAD30::YciR$_{Fec10\ A661T}$) and on pYciR35 (pBAD30::YciR$_{Fec101T37I}$)
YciR-D253E_FW	AATCGTAACGCAATGCAGGA**G**TTAATCGATCACGCTATT	Primer for site-directed mutagenesis on pYciR5 (pBAD30::YciR$_{Fec10\ A661T}$)
YciR-D253E_RV	AATAGCGTGATCGATTAA**C**TCCTGCATTGCGTTACGATT	Primer for site-directed mutagenesis on pYciR5 (pBAD30::YciR$_{Fec10\ A661T}$)
YciR-Fec10_A361S_FW	AGCTGTTCAGTAGGTATT**T**CACTCTCTCCCGAACATGGT	Primer for site-directed mutagenesis on pYciR5 (pBAD30::YciR$_{Fec10\ A661T}$) and on pYciR35 (pBAD30::YciR$_{Fec101T37I}$)
YciR-Fec10_A361S_RV	ACCATGTTCGGGAGAGAGTG**A**AATACCTACTGAACAGCT	Primer for site-directed mutagenesis on pYciR5 (pBAD30::YciR$_{Fec10\ A661T}$) and on pYciR35 (pBAD30::YciR$_{Fec101T37I}$)
YciR-T371A-fw	CGTGACGAATAATAGCCG**C**GCTGTCTGAACCATGT	Primer for site-directed mutagenesis on pYciR5 (pBAD30::YciR$_{Fec10\ A661T}$) and pYciR25 (pBAD30::YciR$_{Fec10\ T37I)}$
YciR-T371A-rv	ACATGGTTCAGACAGC**G**CGGCTATTATTCGTCACG	Primer for site-directed mutagenesis on pYciR5 (pBAD30::YciR$_{Fec10\ A661T}$) and pYciR25 (pBAD30::YciR$_{Fec10\ T37I)}$
YciR-Q552R_FW	GTTATTCAACAATTTAGCC**G**ACTAGGTGCGCAAGTGCAT	Primer for site-directed mutagenesis on pYciR5 (pBAD30::YciR$_{Fec10\ A661T}$)
YciR-Q552R_RV	ATGCACTTGCGCACCTAGT**C**GGCTAAATTGTTGAATAAC	Primer for site-directed mutagenesis on pYciR5 (pBAD30::YciR$_{Fec10\ A661T}$)
yciR-FEC101-GGDAF-mut-f	GGTGGCGATG**C**GTTTCTGGTACTGGCATCC	Primer for site-directed mutagenesis on pYciR3 (pBAD30::YciR$_{Tob1}$) and pYciR7 (pBAD30::YciR$_{Fec101\ A661T}$)
yciR-FEC101-GGDAF-mut-r	AGAAAC**G**CATCGCCACCTGGACGCGC	Primer for site-directed mutagenesis on pYciR3 (pBAD30::YciR$_{Tob1}$) and pYciR7 (pBAD30::YciR$_{Fec101\ A661T}$)

GGAAF-For	GGTGGCG**C**TG**C**GTTTCTGGTACTGGCATCC	Primer for site-directed mutagenesis on pYciR3 (pBAD30::YciR$_{Tob1}$) and pYciR7 (pBAD30::YciR$_{Fec101\ A661T}$)
GGAAF-Rev	AGAAAC**G**CA**G**CGCCACCTGGACGCGC	Primer for site-directed mutagenesis on pYciR3 (pBAD30::YciR$_{Tob1}$) and pYciR7 (pBAD30::YciR$_{Fec101\ A661T}$)
AAL-For	GTCTGG**C**AGCACTAGTACGTTGGCAG	Primer for site-directed mutagenesis on pYciR3 (pBAD30::YciR$_{Tob1}$) and pYciR7 (pBAD30::YciR$_{Fec101\ A661T}$)
AAL-Rev	GTACTAGTGCT**G**CCAGACTGCGCACTTCG	Primer for site-directed mutagenesis on pYciR3 (pBAD30::YciR$_{Tob1}$) and pYciR7 (pBAD30::YciR$_{Fec101\ A661T}$)
ALTE-For	AGATGTTG**C**ACTGACAGAGAGTTGTCTG	Primer for site-directed mutagenesis on pYciR7 (pBAD30::YciR$_{Fec101\ A661T}$)
ALTE-Rev	CTCTGTCAGT**G**CAACATCTATAGGGCAG	Primer for site-directed mutagenesis on pYciR7 (pBAD30::YciR$_{Fec101\ A661T}$)
IKL-IAL-For	TGCCATC**GC**ACTTGACCAGGTTTTTGTTC	Primer for site-directed mutagenesis on pYciR3 (pBAD30::YciR$_{Tob1}$) and pYciR7 (pBAD30::YciR$_{Fec101\ A661T}$)
IKL-IAL-Rev	TGGTCAAGT**GC**GATGGCATCGATCGGAAAG	Primer for site-directed mutagenesis on pYciR3 (pBAD30::YciR$_{Tob1}$) and pYciR7 (pBAD30::YciR$_{Fec101\ A661T}$)
AGVE-for	CCG**C**AGGTGTAGAGAGTGCAAAG	Primer for site-directed mutagenesis on pYciR7 (pBAD30::YciR$_{Fec101\ A661T}$)
AGVE-Rev	CTCTACACCT**G**CGGCGATCACCTGAAG	Primer for site-directed mutagenesis on pYciR7 (pBAD30::YciR$_{Fec101\ A661T}$)

Restriction sites are marked in italics, the 6xHis tag in bold, mutated nucleotides are bold and underlined, priming sites for amplification of antibiotic cassette are underlined.

Table S3: Amino acid changes in c-di-GMP metabolizing proteins compared to the respective *E. coli* K-12 reference protein sequences. Analysis of amino acid substitutions between c-di-GMP metabolizing proteins of the investigated strains. Protein sequences were compared to the respective reference protein from *E. coli* K-12 MG1655 if not otherwise indicated. Protein domain organization is shown for the K-12 reference as predicted by SMART (http://smart.embl-heidelberg.de).

Protein	Strain	Domain structure; exchanges [aa] compared to K-12	comments
**BluF**<br>**YcgF**	K-12<br>MG1655	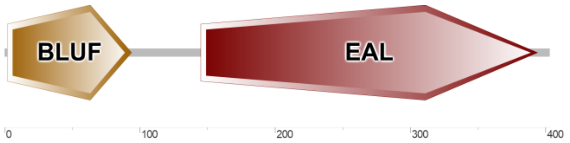	BLUF: 2 - 94<br>***EAL: 145-394***
	Nissle	D13G,Q33R,K59Q,**A165T,P168S,T169N,G212E, T227A,E233K,E251K,N260D,I292V,I349M,A387S,I397V**	
	UTI89	D13G,Q33R,K59Q,**A165T,P168S,T169N,G212E,T227A, E233K,E251K,N260D,I292V,D323N,I349M,A387S,I397V**	additional 26 aa at the N-terminus
	Tob1	D14A,Q33R,K59Q,**A165T,P168S,T169N,D211E,T227A,E233K,N260D,I292V,I349M,A387S,I397V**	
	Fec67	D13G,Q33R,K59Q,**A165T,P168S,T169N,G212E,T227A,E233K,E251K,N260D,I292V,I349M,A387S,I397V**	
	Fec101	-	
	B-11870	D13G,Q33R,K59Q,**A165T,P168S,T169N,G212E, T227A,E233K,E251K,N260D,I292V,I349M,A387S,I397V**	
	No.12	D13G,Q33R,K59Q,**A165T,P168S,T169N,G212E, T227A,E233K,E251K,N260D,I292V,I349M,A387S,I397V**	
	B-8638	S139A,**A165T,T169N,D211E,T227A,E233D,N260D, T333N**	

	Fec10	Stop 57	Stop codon at position 57, giving rise to a truncated protein of only 56 aa. Annotated to restart at aa 59 of K-12 orf
**Protein**	**Strain**	**Domain structure; exchanges [aa] compared to K-12**	**comments**
**CdgI**<br>**YeaI**	K-12<br>MG1655	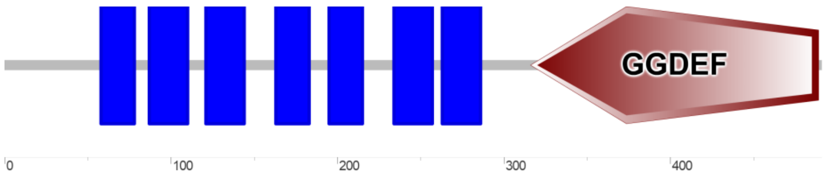	Transmembrane regions:<br>58 - 77; 87 – 109;<br>121 – 143; 163 – 182; 195 – 214; 234 – 256; 263 - 285<br>**GGDEF: 316-489**
	Nissle	Q3R,M9L,K14N,V46A,S57G,**R462H**	
	UTI89	Q3R,M9L,K14N,S57G,**R462H**	
	Tob1	Q3R,M9L,K14N,V46A,S57G,**R462H**	
	Fec67	Q3R,M9L,K14N,S57G**,R462H**	
	Fec101	-	
	B-11870	Q3R,M9L,K14N,P24L,S57G,**R462H**	
	No.12	Q3R,M9L,K14N,P24L,S57G,**R462H**	
	B-8638	Q3R,I7V,M9L,Y28H,V46A,S57G	
	Fec10	L63S, **V354I**	
**Protein**	**Strain**	**Domain structure; exchanges [aa] compared to K-12**	**comments**

**CsrD**<br>**YhdA**	K-12<br>MG1655	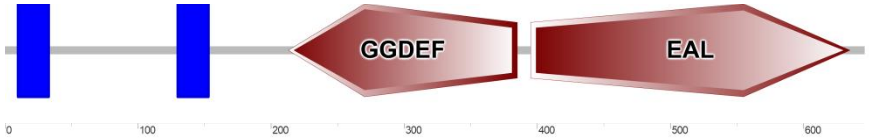 	transmembrane regions:<br>10 -32; 130 – 152<br>**GGDEF: 213-385**<br>***EAL: 395-635***
	Nissle	H52Y,V60T,S100T,S101N,V149L,R204C,**V228M,T284S,G326S,*Q425R,Q464L,K511R***	
	UTI89	V60T,S100T,S101N,V149L,R204C,**V228M,T284S,G326S,*Q425R,Q464L,K511R***	
	Tob1	H52Y,V60T,S100T,S101N,V149L,R204C,**V228M,T284S,G326S,*K511R***	
	Fec67	H52Y,V60T,S100T,S101N,V149L,R204C,**V228M,T284S,G326S,*Q425R,Q464L,K511R***	
	Fec101	V60T	
	B-11870	V60T,S100T,S101N,V149L,R204C,**V228M,T284S,G326S,*Q425R,Q464L,V486A,K511R***	
	No.12	V60T,S100T,S101N,V149L,R204C,**V228M,T284S,G326S,*Q425R,Q464L,V486A,K511R***	
	B-8638	V60A,Q64L,S100T,V149L,**V228M,T284S,G326S,*R473H,K507R***	
	Fec10	-	

**Protein**	**Strain**	**Domain structure; exchanges [aa] compared to K-12**	**comments**
**RflP**<br>**YdiV**	K-12<br>MG1655	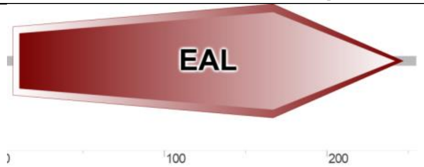 	Catalytically non-functional EAL domain

	Nissle	V24D, E54V, E96D, Y100F, L121F, G129D, V136M, A204P, T211I	
	UTI89	V24D, Q89H, E96D, Y100F, L121F, G129D, V136M, A204P, T211I	
	Tob1	V24D, E54V, E96D, Y100F, L121F, G129D, V136M	Predicted to have a 4 aa truncation at N-terminus, different sequence from aa 200
	Fec67	V24D, Q89H, E96D, Y100F, L121F, G129D, V136M, A204P, T211I	
	Fec101	V24L	
	B-11870	V24D, E54V, E96D, Y100F, L121F, G129D, V136M, A204P, T211I	
	No.12	V24D, E54V, E96D, Y100F, L121F, G129D, V136M, A204P, T211I	
	B-8638	V24D, E54A, E96D, N122D, G129D, V136M, A204P, L207M	
	Fec10	-	
**Protein**	**Strain**	**Domain structure; exchanges [aa] compared to K-12**	**comments**
**AdrA**<br>**YaiC**<br>**DgcC**	K-12<br>MG1655	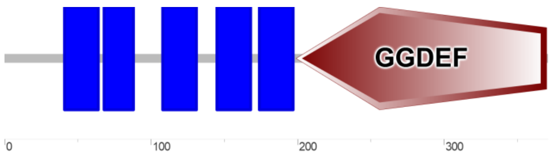	Transmembrane regions:<br>41 – 63; 68 – 87; 108 – 130; 145 – 167; 174 - 196<br><br>**GGDEF: 199-370**
	Nissle	-	
	UTI89	P65S,V72L,V120A	
	Tob1	P65S,V72L,V120A	

	Fec67	P65S,V72L,V120A	
	Fec101	I183V,**H313R**	
	B-11870	S33F,P65S,V72L,V120A	
	No.12	S33F,P65S,V72L,V120A	
	B-8638	-	
	Fec10	-	
**Protein**	**Strain**	**Domain structure; exchanges [aa] compared to K-12**	**comments**
**YegE**<br>**DgcE**	K-12<br>MG1655	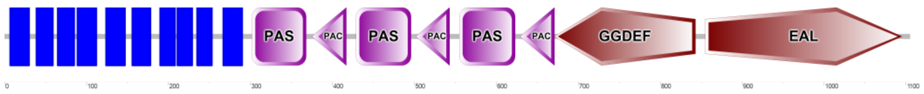	Transmembrane regions:<br>7 -29; 39 – 58; 65 – 84; 89 – 111; 124 – 146; 156 – 177; 190 – 207; 211 - 228; 235 – 252; 267 – 289<br>PAS: 302 – 368, PAC: 375 – 417; PAS: 429 – 496; PAC: 502 -543; PAS: 555 - 623<br>PAC: 629 – 671; **GGDEF: 671–843;** ***EAL: 855-1095***
	Nissle	V134I,H303R,T327S,E412A,T508A,**V842I**,***M857I,L874I, V878I,I883L,R887S,G901S,R924Q,F927C,E929D,I943L,S946A,D962E,P974A,L987F,L1005F,E1085D,D1089N***,S1099R	
	UTI89	V144I,H303R,T327S,***M857I,W863R,L874I,E885Q,E929D,V938I,I943L,S946A,D962E,<br>L964I,E984D,E991A,L1005F,N1035S,E1085D,A1088V***	

	Tob1	V134I,H303R,T327S,**V842I**,***M857I,L874I,V878I,I883L,R887S,G901S,R924Q,F927C,E929D,I943L,S946A,D962E,P974A,L987F,L1005F,M1041I,E1085D,D1089N***	
	Fec67	V134I,H303R,T327S,T508A,**V842I**,***M857I,L874I,P884T,A886V,I903F,R910S,D915E,E929D,S946A,D962E,P974A,L987F,V1008M,M1071I,E1085D,D1089N***,S1099R	
	Fec101	L129F,H303R,**I702M,V774I**, ***L874M,T908K,Q933L,S946A***	
	B-11870	V134I,H303R,T327S,**V842I**,***M857I,L874I,V878I,I883L,R887S,G901S,R924Q,F927C,E929D,I943L,S946A,D962E,P974A,E984D,L987F,L1005F,E1085D,D1089N***,S1099R	Predicted to have a 100 aa truncation at N-terminus
	No.12	V134I,H303R,T327S,**V842I**,***M857I,L874I,V878I,I883L,R887S,G901S,R924Q,F927C,E929D,I943L,S946A,D962E,P974A,E984D,L987F,L1005F,E1085D,D1089N***,S1099R	
	8638	S266T,H303R,T327S,T508A, ***L874M,L922F,S946A,V1004I,E1085D***	
	Fec10	I98M, V141G, H303R, **V774I**, ***L874I, T908K, S946A, E991V, E1085D***	
**Protein**	**Strain**	**Domain structure; exchanges [aa] compared to K-12**	**comments**
**YneF DgcF**	K-12 MG1655	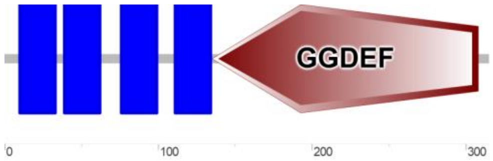	Transmembrane regions:<br>10 – 32; 39 – 61; 76 – 98; 111 -133;<br><br>**GGDEF: 135–308**<br><br>The protein might start 13 AA earlier
	Nissle	I90V,**R206Q,R273C,L277R**	additional 157 aa at N-terminus
	UTI89	I90V,**N136H,R206Q,R273C,L277R**	additional 157 aa at N-terminal end
	Tob1	I90V,**R206Q,R273C,L277R**	additional 157 aa at N-terminal end

	Fec67	I90V,**R206Q,R273C,L277R**	additional 157 aa at N-terminal end
	Fec101	L277R	additional 157 aa at N-terminal end
	B-11870	I90V,**R206Q,R273C,L277R**	additional 104 aa at N-terminal end
	No.12	I90V,**R206Q,R273C,L277R**	additional 157 aa at N-terminal end
	B-8638	**R206Q,R273C,L277R**	additional 157 aa at N-terminal end
	Fec10	-	
**Protein**	**Strain**	**Domain structure; exchanges [aa] compared to K-12**	**Comments**
**YliF**<br>**DgcI**	K-12<br>MG1655	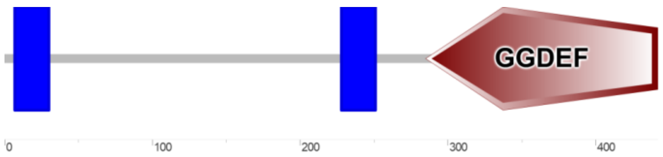 	transmembrane regions:<br>7 – 29; 228 - 250<br>**GGDEF: 285-442**
	Nissle	S37G, P186S, A209S, L226F, R227Q, **P415T**	
	UTI89	S37G, P186S, A209S, L226F, R227Q, **P415T**	
	Tob1	S37G, P186S, A209S, L226F, R227Q, **P415T**	
	Fec67	S37G, P186S, A209S, L226F, R227Q**, P415T**	
	Fec101	N150K, A245T	
	B-11870	S37G, P186S, A209S, L226F, R227Q, **Stop 369, P415T**	aa 369 replaced by stopcodon, orf present again from aa 375 on. Sequence "GGDEFA" is missing.

	No.12	S37G, P186S, A209S, L226F, R227Q, **P415T**	
	B-8638	S37G, V121I, Y192F, A245V, **M291L**	
	Fec10	-	
**Protein**	**Strain**	**Domain structure; exchanges [aa] compared to K-12**	**comments**
**YeaJ**<br>**DgcJ**	K-12<br>MG1655	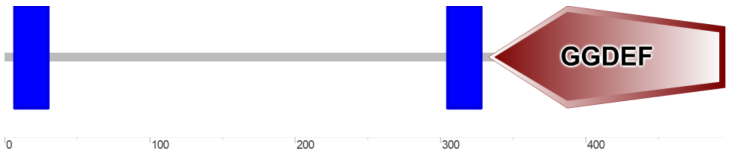	transmembrane regions:<br>7 – 29; 305 - 327<br>**GGDEF: 333-496**
	Nissle	K45R, S76P	
	UTI89	K45R	60 additional aa at the N-terminus
	Tob1	K45R, S76P	
	Fec67	K45R, **T392P**	
	Fec101	**Q388L**	
	B-11870	K45R	
	No.12	L8R, K45R	
	B-8638	K45R	
	Fec10	-	

Protein	Strain	Domain structure; exchanges [aa] compared to K-12	comments
**YdaM**<br>**DgcM**	K-12<br>MG1655	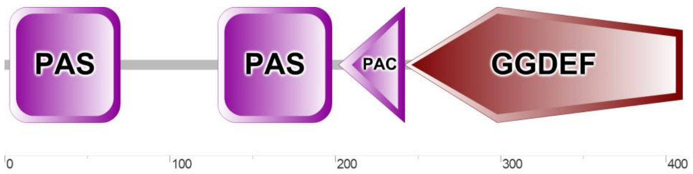 	PAS: 3 – 70<br>PAS: 129 – 198<br>PAC: 202 - 242<br>**GGDEF: 242-410**
	Nissle	K34R, T98A, **G366S**	20 additional aa at N-terminus
	UTI89	K34R, T98A, **G366S**	20 additional aa at N-terminus
	Tob1	K34R, K80N, T98A, V206I, **G366S**	
	Fec67	K34R, K94E	
	Fec101	K34R, K94E	
	B-11870	K34R, T98A, **G366S**	
	No.12	K34R, T98A, **G366S**	
	B-8638	K34R, K94E, T98A, L136R	
	Fec10	-	
**Protein**	**Strain**	**Domain structure; exchanges [aa] compared to K-12**	**comments**
**YfiN**<br>**TpbB**<br>**DgcN**	K-12<br>MG1655		Transmembrane regions:<br>23 – 45; 155 - 177<br>**GGDEF: 238-408**

		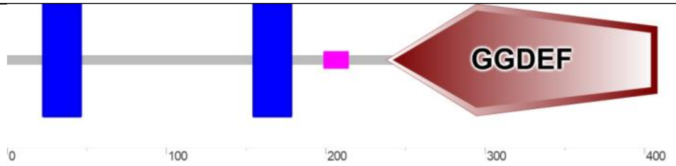	
	**strain**	**aa exchanges compared to K-12**	
	Nissle	R109H, D111E, **K386N**	
	UTI89	R109H, D111E, **K386N**	
	Tob1	N9H, M31T, R109H, D111E, **K386N**	
	Fec67	N9H, M31T, R109H, D111E, **K386N**	
	Fec101	-	
	B-11870	N9H, M31T, R109H, D111E, **K386N**	
	No.12	N9H, M31T, R109H, D111E, **K386N**	
	B-8638	G165A	
	Fec10	-	
**Protein**	**Strain**	**Domain structure; exchanges [aa] compared to K-12**	**comments**
**YddV**<br>**DosC**<br>**DgcO**	K-12<br>MG1655		Globin sensor domain: 7-155<br>**GGDEF: 283 - 456**

		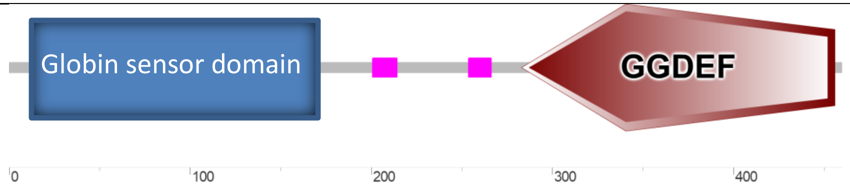	
	Nissle	**W456C**	Truncated N-terminus, matches orf from aa 407; exhibiting 16 preceeding aa.
	UTI89	**W456C**	Truncated N-terminus, matches orf from aa 407; exhibiting 18 preceeding aa.
	Tob1		Not encoded in chromosome
	Fec67		Not encoded in chromosome
	Fec101	P22L, V32L	
	B-11870		Not encoded in chromosome
	No.12		Not encoded in chromosome
	B-8638	P22L, I200V	
	Fec10	-	
**Protein**	**Strain**	**Domain structure; exchanges [aa] compared to K-12**	**comments**
**YeaP**<br>**DgcP**	K-12<br>MG1655	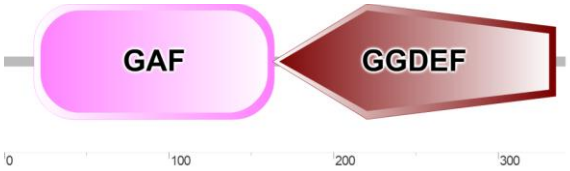	GAF: 18-164<br>**GGDEF: 163-335**

	Nissle	Y63H, **A335T, P337S**	43 additional aa at the N terminus
	UTI89	Y63H, I99V**, A335T, P337S**	43 additional aa at the N terminus
	Tob1	Y63H, I99V**, A335T, P337S**	
	Fec67	Y63H, I99V**, A335T, P337S**	
	Fec101	Y63H	
	B-11870	Y63H, I99V, **A335T, P337S**	
	No.12	Y63H, I99V, **A335T, P337S**	
	B-8638	Y63H	
	Fec10	-	
**Protein**	**Strain**	**Domain structure; exchanges [aa] compared to K-12**	**comments**
**DgcQ**<br>**YedQ**	K-12<br>MG1655	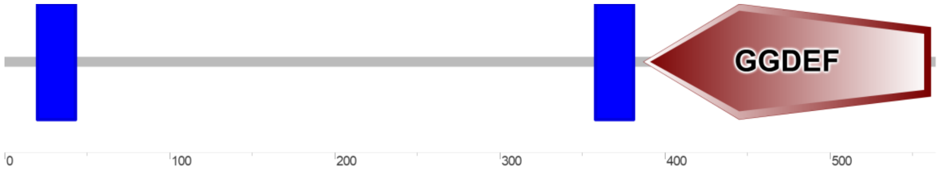	Transmembrane regions:<br>20-42; 358-380<br>**GGDEF: 387-561**
	Nissle	V25I, D100N, K115Q, M175V, Q187L, T371S, I375L	Del aa 1-6, from aa 407: different sequence, GGDEF domain absent
	UTI89	V25I, D100N, K115Q, D325N, **E492Q, F559C**	5 additional aa at the N terminus
	Tob1	T96I, D100N, K115Q, T371S, I375L, **R417C, Q424H, V472I, S489N, E492Q**	
	Fec67	T96I, D100N, K115Q, T371S, I375L, **R417C, Q424H, V472I, S489N, E492Q**	

	Fec101	**L422M**	
	B-11870	V25I, D100N, K115Q, M175V, Q187L, T371S, I375L, **R417C, Q424H, V472I, S489N, E492Q**	
	No.12	D100N, K115Q, M175V, Q187L, I375L, **S489N, E492Q**	
	B-8638	D100N, K115Q, F288L, P297S, H316Q, D325N	
	Fec10	K115Q, P125S, S167T, D335N	
**Protein**	**Strain**	**Domain structure; exchanges [aa] compared to K-12**	**comments**
**YcdT**<br>**DgcT**	K-12<br>MG1655	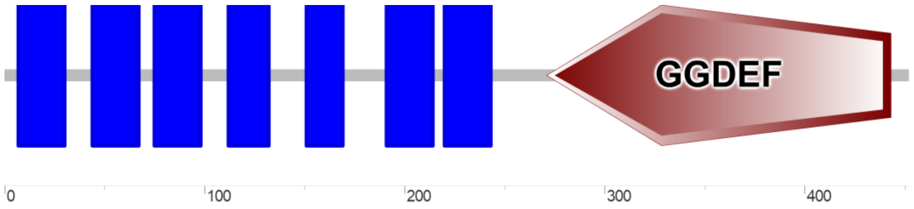	Transmembrane regions:<br>7-29; 44-66; 75-97; 112-131; 151-168; 191-213; 220-242<br>**GGDEF: 271-443**
	Nissle	R7G, G33T, V76I, V89I, I90K, E101G, F159V, G187D, Y218C, I236V, T248I, **N280D, P286S, Q308K, N343S, L355F, E413K, A415E, N419K, R423Q**	Last 10 aa exchanges against 12 aa stretch KEAEILIRKDDE
	UTI89	R7G, G33T, V76I, V89I, I90K, E101G, F159V, G187D, A193T, Y218C, I236V, T248I, **N280D, P286S, Q308K, N343S, L355F, E413K, A415E, N419K, R423Q**	41 additional aa at the N terminus, last 10 aa exchanges against 12 aa stretch KEAEILIRKDDE
	Tob1		Not encoded in chromosome
	Fec67	R7G, G33T, V76I, V89I, I90K, E101G, I125V, M149V, S154T, F159L, P162A, L168F, N176D, Y218C, T225S, T248I, M264L, **N280D, Q308K, D369E, R374C, E413K, A415E, N419K, R423Q**	Last 10 aa exchanges against 12 aa stretch KEAEILIRKDDE
	Fec101		Not encoded in chromosome

	B-11870	R7G, G33T, V76I, V89I, I90K, E101G, I125V, M149V, S154T, F159L, L168F, N176D, E185K, Y218C, T225S, T248I, M264L, **N280D, Q308K, D369E, R374C, E413K, A415E, N419K, R423Q**	Last 10 aa exchanges against 12 aa stretch KEAEILIRKDDE
	No.12	R7G, G33T, V76I, V89I, I90K, E101G, I125V, M149V, S154T, F159L, P162S, L168F, N176D, E185K, Y218C, T225S, T248I, M264L, **N280D, Q308K, D369E, R374C, E413K, A415E, N419K, R423Q**	Last 10 aa exchanges against 12 aa stretch KEAEILIRKDDE
	B-8638		Not encoded in chromosome
	Fec10	-	
**Protein**	**Strain**	**Domain structure; exchanges [aa] compared to K-12**	**comments**
**YdeH**<br>**DgcZ**	K-12<br>MG1655	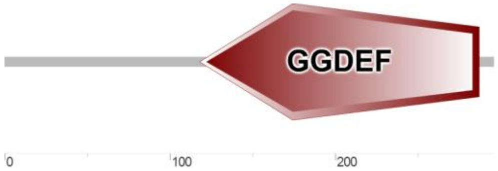	**GGDEF: 118-287**
	Nissle	**N130S, A217T**	
	UTI89	H31R, **N130S, A217T**	2 additional aa at N-terminus
	Tob1	**N130S, A217T**	
	Fec67	R56W, **N130S, A217T**	
	Fec101	-	
	B-11870	H31R	
	No.12	H31R	

	B-8638	I58L, D104G	
	Fec10	-	
**Protein**	**Strain**	**Domain structure; exchanges [aa] compared to K-12**	**comments**
**YciR**<br>**PdeR**<br>**Gmr**	K-12<br>MG1655	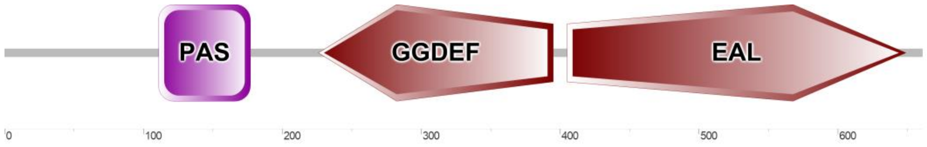 	PAS: 111-177<br>**GGDEF: 226-395**<br>***EAL: 405-649***
	Nissle	T37I, T43I, A90G, C192R, **D253E, H261Q, M333L, A361S, T371A**, ***Q552R***	
	UTI89	T37I, T43I, A90G, C192R, **D253E, H261Q, M333L, A361S, T371A**, ***Q552R,*** A661T	
	Tob1	T37I, T43I, A90G, V156M, C192R, **D253E, H261Q, A361S, T371A,** ***Q552R***, A661T	
	Fec67	T8A, T37I, T43I, A90G, C192R, **D253E, H261Q, M333L, A361S, T371A,** ***Q552R***	
	Fec101	C192R	
	B-11870	T37I, T43I, A90G, C192R, **D253E, H261Q, M333L, A361S, T371A,** Stop 432, ***Q552R,*** A661T	Stop codon replacing codon 432; next Orf corresponding to YciR starting at aa 436
	No.12	T37I, T43I, A90G, C192R, **D253E, H261Q, M333L, A361S, T371A, Q552R**, A661T	
	B-8638	D41E, C192R, **D253E, H261Q, A361S, T371A,** ***A622K***	
	Fec10	-	

Protein	Strain	Domain structure; exchanges [aa] compared to K-12	comments
**YfeA**<br>**PdeA**	K-12<br>MG1655	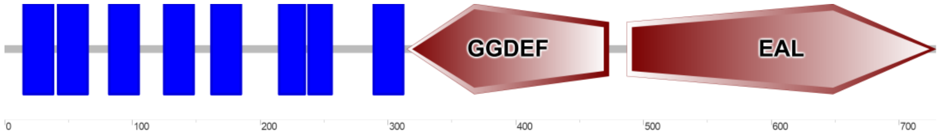	transmembrane regions:<br>15-37; 42-64; 82-104; 125-147; 162-184; 215-234; 238-255; 289-311<br>**GGDEF: 315-473**<br>***EAL: 487-729***
	Nissle	F30L, I36V, S40N, V67I, F79L, L90I, T94A, V96A, T132A, S145N, G209S, L230M, L258I, E286L, **M370I, C380Y, *H601Y, Q702P***	
	UTI89	F30L, I36V, S40N, V67I, F79L, L90I, T94A, V96A, T132A, S145N, G209S, L230M, L258I, E286L, **M370I, C380Y, *H601Y, Q702P***	20 additional aa at N terminus
	Tob1	F30L, I36V, S40N, V67I, F79L, L90I, T94A, V96A, T132A, S145N, G209S, L230M, L258I, E286L, **M370I, C380Y, *H601Y, Q702P***	
	Fec67	F30L, I36V, S40N, V67I, F79L, L90I, T94A, V96A, T132A, S145N, G209S, L230M, L258I, E286L, **M370I, C380Y, *H601Y***	
	Fec101	***D520E***	
	B-11870	F30L, I36V, S40N, V67I, F79L, L90I, T94A, V96A, T132A, S145N, G209S, L230M, L258I, E286L, **M370I, C380Y, *H601Y, Q702P***	
	No.12	F30L, I36V, S40N, V67I, F79L, L90I, T94A, V96A, T132A, S145N, G209S, L230M, L258I, E286L, **M370I, C380Y, *H601Y, Q702P***	
	B-8638	G209S, E286L, **C380Y**	
	Fec10	-	

Protein	Strain	Domain structure; exchanges [aa] compared to K-12	comments
**YjcC**<br>**PdeC**	K-12<br>MG1655	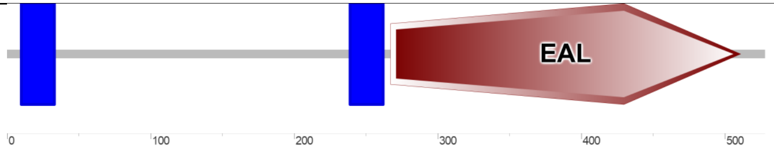	transmembrane regions:<br>10-32<br>239-261<br>***EAL: 267-511***

	Nissle	L12F, L25M, L32F, P112S, K189N, K203N, ***I315V, I334V, D348A, A354T, I376T, Y383H, Q414K, E485A***, E517G	11 aa shorter at the C-terminus
	UTI89	L12F, L32F, P112S, K189N, K203N***, I315V, I334V, D348A, A354T, I376T, Y383H, Q414K, E485A***	3 aa additional at the N-terminus
	Tob1	L12F, L25M, L32F, P112S, K189N, K203N***, I315V, I334V, D348A, A354T, I376T, Y383H, Q414K, E485A***, E517G	11 aa shorter at the C-terminus
	Fec67	L12F, P13S, L32F, P112S, K189N, K203N, ***I315V, I334V, D348A, A354T, I376T, Y383H, Q414K, E485A, R493H***, E517G	11 aa shorter at the C-terminus
	Fec101	***P309L, D403E***	After aa 479: different sequence
	B-11870	L12F, L32F, P112S, K189N, K203N***, I315V, I334V, D348A, A354T, I376T, Y383H, Q414K, E485A, R493H***, E517G	11 aa shorter at the C-terminus
	No.12	L12F, L32F, P112S, K189N, K203N***, I315V, I334V, D348A, A354T, I376T, Y383H, Q414K, E485A, R493H***, E517G	11 aa shorter at the C-terminus
	B-8638	R4H, N40S, R43Q, , K189N, K203N, T237A, ***I300T, L306I, I315V, D348V, A354V, I376T***	
	Fec10	-	
**Protein**	**Strain**	**Domain structure; exchanges [aa] compared to K-12**	**comments**
**YoaD**<br>**AdrB**<br>**PdeD**	K-12<br>MG1655	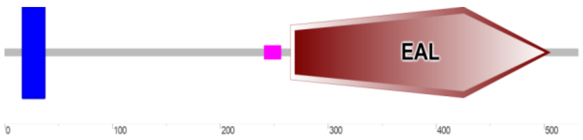	transmembrane regions:<br>17-36<br>***EAL: 265-506***
	Nissle	A82T, I138T, K193S, D211N, ***G464A, R487S***	
	UTI89	I138T, K193S, D211N, R505C	10 additional aa at N terminus
	Tob1	I138T, R140H, S159N, N160K, T173A, K193N, D211N	

	Fec67	I138T, K193S, D211N, ***R487S***	
	Fec101	-	
	B-11870	I138T, K193S, D211N	
	No.12	I138T, K193S, D211N	
	B-8638	A53T	
	Fec10	-	
**Protein**	**Strain**	**Domain structure; exchanges [aa] compared to K-12**	**comments**
**YlaB**<br>**PdeB**	K-12<br>MG1655	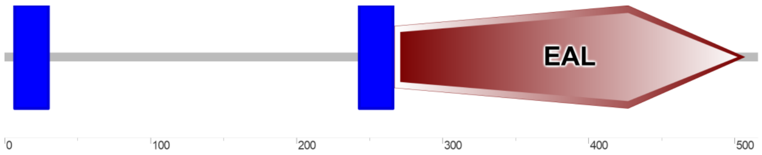	transmembrane regions: 7-29; 243-265<br><br>***EAL: 267-507***
	Nissle	G8S, D42N, A96V, N136S, E190K, ***P365T, D378E***	
	UTI89	D42N, A96V, I191L, ***P365T, D378E, E463K***	2 additional aa at N terminus
	Tob1	G8S, D42N, A96V, E190K, ***P354S, P365T, D378E***	
	Fec67	D42N, A96V, E190K, ***P354S, P365T, D378E***	
	Fec101	A96V, L219F, ***P365T, D378E***	
	B-11870	D42N, A96V, D99N, E190K, T197A, G238S, ***P354S, P365T, D378E***	

	No.12	D42N, A96V, D99N, E190K, T197A, G238S, ***P354S, P365T, D378E***	
	B-8638	D42N, A96V, L219F, ***P365T, D378E***	
	Fec10	-	
**Protein**	**Strain**	**Domain structure; exchanges [aa] compared to K-12**	**comments**
**YfgF**<br>**PdeF**	K-12<br>MG1655	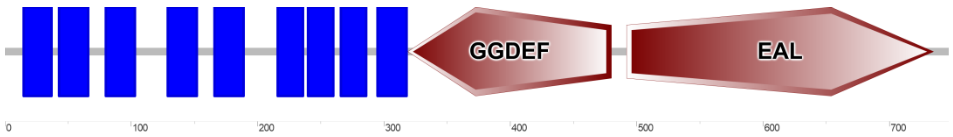	transmembrane regions<br>15-36; 43-65; 80-102; 129-151; 166-188; 216-235; 240-259; 266-285; 295-317<br>**GGDEF: 319-480**<br>***EAL: 492-735***
	Nissle	T118S, I125L, L218I, ***Q502R, L699R***	
	UTI89	T118S, I125L, L218I, ***Q502R, L699R***	
	Tob1	T118S, I125L, L218I, ***Q502R, L699R***	
	Fec67	T118S, I125L, Y200S, L218I, ***Q502R, L699R***	
	Fec101	A69T, ***S540G, L699R***	
	B-11870	T118S, I125L, L218I, ***Q502R, L699R***	
	No.12	T118S, I125L, L218I, ***Q502R, L699R***	
	B-8638	S61R, A209V, V211A, ***L699R***	
	Fec10	-	

Protein	Strain	Domain structure; exchanges [aa] compared to K-12	comments
**YcgG**<br>**PdeG**	K-12<br>MG1655	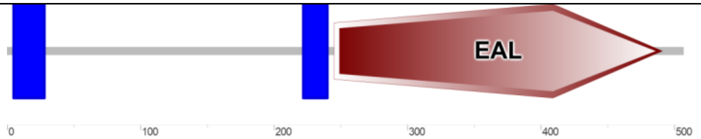	transmembrane regions:<br>5-27<br>222-239<br>***EAL: 245-491***
	Nissle	***S306C, S348L, F358Y, E437A, G460D, L467F, Y474N,*** V504I	224 aa shorter at N terminus
	UTI89		Not encoded in chromosome
	Tob1	***A298V, S306C, F358Y, E437A, L467F, Y474N***	222 aa shorter; 22 aa nonmatching at N-terminus, starting to match reference from aa 245
	Fec67	***S306C, S348L, F358Y, E437A, G460D, L467F, Y474N,*** V504I	224 aa shorter at N terminus
	Fec101	***Y474N***	
	B-11870	***S306C, F358Y, E437A, L467F, Y474N,*** V504I	224 aa shorter at N terminus
	No.12	***S306C, F358Y, E437A, L467F, Y474N***, V504I	224 aa shorter at N terminus
	B-8638	L9F, V10I, A20T, N68D, I95T, N138S, ***F358Y, Y474N***	
	Fec10	***Y474N***	

Protein	Strain	Domain structure; exchanges [aa] compared to K-12	comments

**YhjH**<br>**PdeH**	K-12<br>MG1655	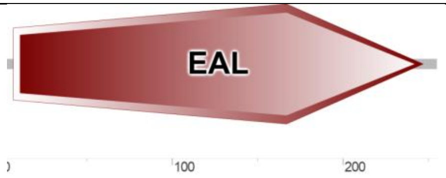	***EAL: 7-247***
	Nissle	***I96V***	
	UTI89	***I96V, R126D***	Double methionine at start
	Tob1	***I96V, R126D***	
	Fec67	***I96V***	
	Fec101	**-**	
	B-11870	***I96V, R126D***	
	No.12	***I96V, R126D***	
	B-8638	***V52L, G99D***	
	Fec10	-	
**Protein**	**Strain**	**Domain structure; exchanges [aa] compared to K-12**	**comments**
**YliE**<br>**PdeI**	K-12<br>MG1655	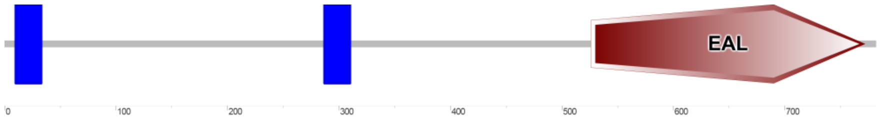	transmembrane regions:<br>10-32<br>287-309<br>***EAL: 526-772***
	Nissle	D116N, V151M, Q209H, E318D, S364A, M462T, W492R, D507N, ***A557T, I682V***	

	UTI89	V151M, Q209H, E318D, S364A, M462T, D507N, ***A557T, I682V***	
	Tob1	V151M, Q209H, E318D, S364A, M462T, D507N, ***A557T, I682V***	
	Fec67	N31D, V151M, Q209H, E318D, S364A, M462T, D507N, ***A557T, Q610E, S613V, D617G, I682V, I700M***	
	Fec101	H180R, E257A, L303F	
	B-11870	N31D, V151M, Q209H, E318D, S364A, M462T, D507N, **A557T, I682V**	
	No.12	N31D, V151M, Q209H, E318D, S364A, M462T, D507N, ***A557T, I682V***	
	B-8638	Q125E, H180R, A261V, V463I, ***A649E***	
	Fec10	-	
**Protein**	**Strain**	**Domain structure; exchanges [aa] compared to K-12**	**comments**
**YhjK**<br>**HmsP**<br>**PdeK**	K-12<br>MG1655	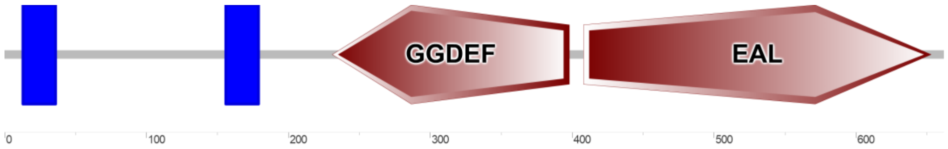	transmembrane regions:<br>13-35<br>156-178<br>**GGDEF: 231-398**<br>***EAL: 408-653***
	Nissle	A198P, K199Q, **M345I,** ***P400L, G599E***	
	UTI89	A198P, K199Q, ***K408Q, G599E***	
	Tob1	K199Q, ***K408Q, G599E***	Annotated to start 13 aa later
	Fec67	A198P, K199Q, ***G599E***	Annotated to start 13 aa later

	Fec101	K199Q, ***G599E,*** K662R	Annotated to start 13 aa later
	B-11870	A198P, K199Q, **M345I, *G599E***	
	No.12	A198P, K199Q, **M345I**, ***G599E***	Annotated to start 13 aa later
	B-8638	K199Q, ***K408Q, G599E***	Annotated to start 13 aa later
	Fec10	-	
**Protein**	**Strain**	**Domain structure; exchanges [aa] compared to K-12**	**comments**
**YahA<br>PdeL**	K-12<br>MG1655	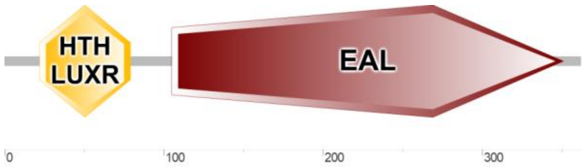	***EAL: 105-351***
	Nissle	S93N, R97K***, I153M, N220K***	
	UTI89	S93N, R97K, ***I153M, N220K***	2 additional aa at N terminus
	Tob1	S93N, R97K, ***I153M, N220K***	
	Fec67	S93N, R97K, ***I153M, N220K***	
	Fec101	***N220K***	
	B-11870	S93N, R97K, ***I153M, N220K***	
	No.12	S93N, R97K, ***I153M, N220K***	

	B-8638	S93N, R97K, ***N220K***	
	Fec10	-	
**Protein**	**Strain**	**Domain structure; exchanges [aa] compared to K-12**	**comments**
**Rtn**<br>**PdeN**	K-12<br>MG1655	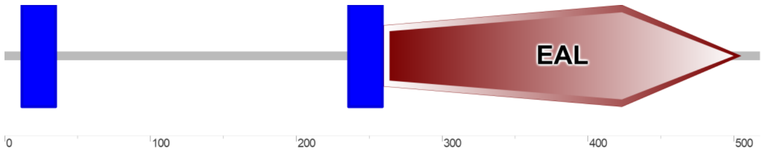 	Transmembrane regions:<br>12-34; 236-258<br>***EAL: 260-505***
	Nissle	F8S, V41A, V185L, V249F, ***A280V, S320A, D359A, Q397R***	
	UTI89	F8S, K42T, V185L, V249F, ***A280V, S320A, D359A, Q397R***	
	Tob1	F8S, V185L, V249F, ***A280V, S320A, D359A, Q397R***	
	Fec67	F8S, N118K, V249F, ***A280V, S320A, D359A, Q397R***	
	Fec101	F8S	
	B-11870	F8S, V185L, V249F, ***A280V, S320A, D359A, Q397R***	
	No.12	F8S, V185L, V249F, ***A280V, S320A, D359A, Q397R***	
	B-8638	F8S, ***A289S***	
	Fec10	-	

**Protein**	**Strain**	**Domain structure; exchanges [aa] compared to K-12**	**comments**
**YddU**<br>**DosP**<br>**PdeO**	K-12<br>MG1655	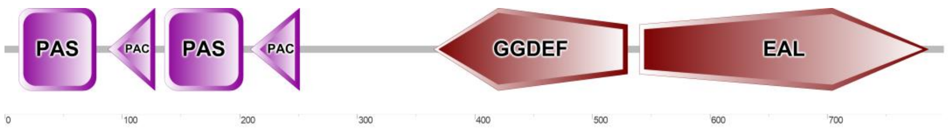	PAS: 12-78; PAC: 88-128; PAS: 136-203; PAC: 209-251<br>**GGDEF: 365-530**<br>***EAL: 540-786***
	Nissle	E71Q, S174N, S284C, I313V, **T453A, Q454S, L501E, Y518F, *H582Y, V692I, V756I, R766H***	
	UTI89	N8T, I101V, S174N, I313V, **T453A, Q454S, L501E, *Y518F, H582Y, V692I, V756I***	8 additional aa at N terminus
	Tob1	E71Q, S174N, I313V, A329T, **T453A, Q454S, L501E, Y518F, *H582Y, V692I, V756I***	
	Fec67	E71Q, S174N, I313V, **Q454S, L501V, Y518F, *H582Y, V692I, V756I***	
	Fec101	I313V, **D396E, D486N, L501V**	
	B-11870	E71Q, S174N, <u>Stop 370</u><br>**V402I, T453A, Q454S, A471S, L501E, Y518F, *H582Y, V692I, V756I***	stop codon after codon 369; from aa 245 to the stop, no resemblance of the K-12 sequence. The ORF restarts at codon 381. Thus, the truncated protein contains the PAS-PAC domains only and potentially a second protein is present starting from the GGDEF domain.
	No.12	E71Q, S174N, I313V, **V402I, T453A, Q454S, A471S, L501E, Y518F, *H582Y, V692I, V756I***	
	B-8638	V116I, A311T, I313V, **L501V, Y518F**	
	Fec10	-	annotated as Heme-regulated cyclic AMP phosphodiesterase in RAST server
**Protein**	**Strain**	**Domain structure; exchanges [aa] compared to reference**	**comments**

**DgcX**	*E. coli* 55989	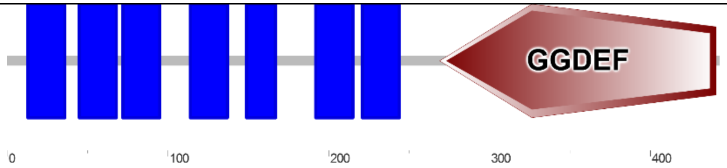	Transmembrane regions:<br>13-35; 45-67; 72-94; 114-136; 149-166; 192-214; 221-243; 269-441<br>**GGDEF: 269-441**
	Nissle		Not encoded in chromosome
	UTI89		Not encoded in chromosome
	Tob1		Not encoded in chromosome
	Fec67		Not encoded in chromosome
	Fec101	Q5K, I8K, I12E, L16I, C19F, I20L, L25M, N30K, Y34H, L50I, I57V, L99I, I106T, V132I, V142I, D145E, V152I, V153T, T194I, Q245K, S246P, H281Y, D309S, E350D, T369S, K399Q, L414H, T440I	
	B-11870		Not encoded in chromosome
	No.12		Not encoded in chromosome
	B-8638		Not encoded in chromosome
	Fec10		Not encoded in chromosome
**Protein**	**Strain**	**Domain structure; exchanges [aa] compared to reference**	**comments**
**PdeX**	UPEC-156	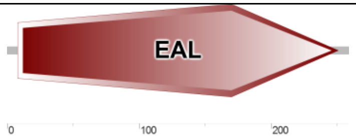	***EAL: 8-251***
	Nissle		Not encoded in chromosome

	UTI89		Not encoded in chromosome
	Tob1	***L102V, T105A, C151F***	
	Fec67		Not encoded in chromosome
	Fec101		Not encoded in chromosome
	B-11870		Not encoded in chromosome
	No.12		Not encoded in chromosome
	B-8638		Not encoded in chromosome
	Fec10		Not encoded in chromosome
**Protein**	**strain**	**Domain structure; exchanges [aa] compared to reference**	**comments**
**PdeY**	UTI89	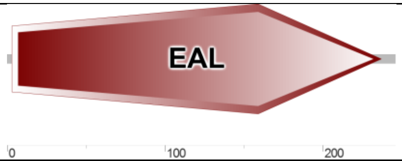	***EAL: 3-237***
	Nissle	***D29N, S62P, P187S, T195S, Y199H,*** I242T	
	Tob1		Not encoded in chromosome
	Fec67	Hit1: ***C184S, Y199H, E234G,*** I242T, L244Q, H245Y<br>Hit2: -	Double hit, both lacking N-terminal part up to aa 159.
	Fec101		Not encoded in chromosome
	B-11870	***D29N, S62P, C184S, Y199H, E234G,*** I242T	

	No.12	***D29N, S62P, C184S, Y199H, E234G,*** I242T	
	B-8638		Not encoded in chromosome
	Fec10		Not encoded in chromosome
**Protein**	**strain**	**Domain structure; exchanges [aa] compared to reference**	**comments**
**PdeU1** (Q4FBC2)	*E. coli*	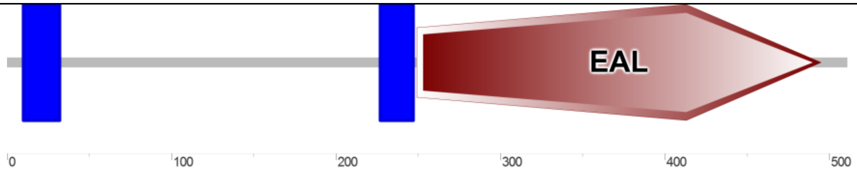	Transmembrane regions:<br>10-31; 227-246<br>***EAL: 249-495***
	Nissle		Not encoded in chromosome
	UTI89		Not encoded in chromosome
	Tob1		Not encoded in chromosome
	Fec67		Not encoded in chromosome
	Fec101		Not encoded in chromosome
	B-11870	-	
	No.12		Not encoded in chromosome
	B-8638		Not encoded in chromosome
	Fec10		Not encoded in chromosome

**Protein**	**strain**	**Domain structure; exchanges [aa] compared to reference**	**comments**
**PdeU2** (Q6EMD2)	*E. coli*	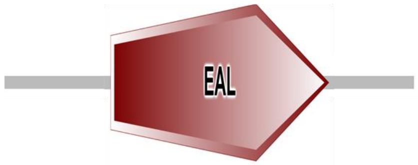	**Non-functional, degenerated EAL domain**
	Nissle		Not encoded in chromosome
	UTI89		Not encoded in chromosome
	Tob1		Not encoded in chromosome
	Fec67		Not encoded in chromosome
	Fec101		Not encoded in chromosome
	B-11870	-	N-terminally shorted, starts at aa 29 of reference orf.
	No.12		Not encoded in chromosome
	B-8638		Not encoded in chromosome
	Fec10		Not encoded in chromosome
**Protein**	**strain**	**Domain structure; exchanges [aa] compared to reference**	**comments**

**PdeU3** (V0Y095)	*E. coli* 908541	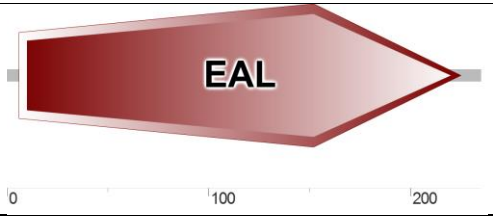	***EAL: 6-225***
	Nissle		Not encoded in chromosome
	UTI89		Not encoded in chromosome
	Tob1		Not encoded in chromosome
	Fec67		Not encoded in chromosome
	Fec101		Not encoded in chromosome
	B-11870		Not encoded in chromosome
	No.12		Not encoded in chromosome
	B-8638	-	
	Fec10		Not encoded in chromosome

84
